# Variations in definitions used for describing restrictive care practices (seclusion and restraint) in adult mental health inpatient units: a systematic review and content analysis

**DOI:** 10.1007/s00127-024-02739-6

**Published:** 2024-07-30

**Authors:** Zelalem Belayneh Muluneh, Jacinta Chavulak, Den-Ching A. Lee, Melissa Petrakis, Terry P. Haines

**Affiliations:** 1 School of Primary and Allied Health Care, Faculty of Medicine, Nursing and Health Sciences, Monash University, Melbourne, Australia; 2 College of Health and Medical Sciences, Department of Psychiatry, Dilla University, Dila, Ethiopia; 3 Rehabilitation, Ageing and Independent Living (RAIL) Research Centre, Monash University, Frankston, VIC Australia; 4 Mental Health Service, St Vincent’s Hospital, Melbourne, Australia; 5 National Centre for Healthy Ageing, Peninsula Health and Monash University, Frankston, VIC Australia

**Keywords:** Restraint, Restrictive practice, Coercion, Immobilization, Seclusion, Mental health, Definition, Understanding, Perception, Classification

## Abstract

**Purpose:**

The main purpose of this review was to (1) identify thematic elements within definitions used by recently published literature to describe the constructs of physical/mechanical restraint, seclusion and chemical restraint in adult mental health inpatient units.

**Methods:**

We conducted a comprehensive literature search of six databases (Scopus, MEDLINE, PsycINFO, Web of Science, Embase, and CINAHL-Plus). In this review, we conducted content analysis to synthesize evidence to understand and compare the commonalities and discrepancies in conceptual elements that were incorporated within the definitions of different forms of restrictive care practices.

**Results:**

A total of 95 studies that provided definitions for different forms of restrictive care practices [physical/mechanical restraint (*n* = 72), seclusion (*n* = 65) and chemical restraint (*n* = 19)] were included in this review. Significant variations existed in the conceptual domains presented within the applied definitions of physical/mechanical restraint, seclusion, and chemical restraint. Conceptual themes identified in this review were methods of restrictive care practice, reasons and desired outcomes, the extent of patient restriction during restrictive care practice episodes, timing (duration, frequency, and time of the day), the level of patient autonomy, and the personnel implementing these practices.

**Conclusions:**

Inconsistencies in the terminologies and conceptual boundaries used to describe the constructs of different forms of restrictive care practices underscore the need to move forward in endorsing consensus definitions that reflect the diverse perspectives, ensuring clarity and consistency in practice and research. This will assist in validly measuring and comparing the actual trends of restrictive care practice use across different healthcare institutions and jurisdictions.

**Supplementary Information:**

The online version contains supplementary material available at 10.1007/s00127-024-02739-6.

## Introduction

Restrictive care practices (RCPs) such as physical/mechanical restraint, seclusion and chemical restraint have often been used in mental health settings to manage patient behaviour and minimize the perceived risk of danger. The recognition of adverse physical and psychological consequences [1, 2] associated with the use of RCPs is growing, leading to an international consensus to reduce or eliminate (when possible) the use of these practices [3, 4]. The use of RCPs may lead to falls [36], bedsores, injuries, immobility, drug reactions (in cases of chemical restraint), decreased physiological well-being and death in extreme cases [37, 38]. The adverse effects of these practices extend beyond patients, affecting family members, caregivers, and clinicians as well [5]. Observing a loved one being subjected to seclusion or restraint can be highly traumatic [6]. Family members and staff often experience a sense of helplessness [5], neglect, and distress [7], affecting the therapeutic environment and delaying the person’s recovery journey [8].

Different policy strategies, guidelines, and intervention practices have been introduced to minimize RCP use and seek less restrictive alternative strategies in mental health care settings [9, 10]. One approach to evaluating the effectiveness of reduction strategies and understanding the trends in the implementation of these practices is comparing prevalence rates of RCP use in similar groups of patients or settings [16, 17]. However, there are considerable variations in the reported rates of restraint and seclusion use from different reduction measures and policy practices [11, 12], making it difficult to understand the clear impacts of these strategies and determine which strategies are more effective in reducing RCP use [13].

Valid comparisons and reliable measurements of the actual practices could only be possible when there are clear and common definitions of RCPs [14]. Studies that used different definitions may involve vastly different measurement outcome reports while the actual practice may not vary as such [15, 16]. For example, the reported prevalence rates of physical restraint for a study that defines physical restraint as “*the use of any physical/manual or mechanical devices*” could differ from a study that defines physical restraint as “*holding the person’s arm*”. The first definition is inclusive of several restraint techniques that can be applied using either human force or equipment devices, while the second one has a strict definition and specifically measures only physical holding. Studies that use broader definitions are more likely to result in a higher measurement outcome (prevalence rate) than studies that use strict definitions [15, 16].

Without clear and common definitions of RCPs, there is a risk of subjective interpretation and inconsistent implementation and understanding, potentially leading to confusion and varied outcomes in clinical settings [16, 17]. Individual interpretations regarding the concept of RCPs can be affected by the involvement of multiple factors such as personal values, professionals’ training level and skills, work experience, differences in cultural norms and policy variations. Such variations can create ethical and legal dilemmas among clinicians [14] when making decisions about whether an action is a restrictive practice [17] and whether it should be documented and reported in the incident reporting system, limiting accurate appraisal of the data collected in mental health settings [18].

Subjectivities surrounding the understanding of RCPs negatively affect contemporary efforts to genuinely reduce the use of these practices and apply least restrictive options [14]. In the absence of clear and consistent definitions of RCPs, healthcare organizations and jurisdictions may have different laws and policies that can fit with their option of interest [19], resulting in disparate approaches [20] to manage, define and record RCP episodes [21]. This creates the possibility that one method of RCP (that falls within their definitions of RCP) may be unintentionally replaced by another method (that is outside their definitions of RCP) but may be even more coercive, to comply with the least restrictive options [22, 23]. Without clear and consensus definitions, researchers and other stakeholders may also use different approaches and indicators for monitoring and evaluating RCP reduction strategies and policy practices, creating discrepancies between the reported data and the actual practice [24, 25]. This makes it challenging to compare findings across studies, assess the effectiveness of interventions, and identify best practices that can effectively minimize these practices and allow for consistently managing patient care in mental health settings.

Attempts have been made to harmonize standards in the definition, implementation, and documentation of RCPs [38]. However, even with uniform definitions, the broader political and legislative environment can shape how these practices are defined and implemented on the ground [18, 19]. These practices are not still yet consistently regulated and explicitly defined in mental health sectors of different regions. For instance, in Finland, the general guidelines allow psychiatric hospitals to develop their own local seclusion policies. However, this has resulted in a lack of uniformity. Similarly, in Australia and New Zealand, the Mental Health Acts vary between states/territories, leading to different seclusion and restraint practices within the same country. The United States, on the other hand, has relatively uniform regulations. However, evidence shows a wide distribution of seclusion and restraint episodes, suggesting issues with their implementation in the clinical practice. Therefore, it is necessary to not only establish standardized terminologies and definitions but also harmonize policies, oversight mechanisms, and implementation strategies across different jurisdictions.

Systematically reviewing existing definitions and identifying the thematic elements incorporated within definitions of RCPs used in recent studies would be the initial step to move towards towards establishing standardized definitions and consistent policy frameworks [30]. However, there is a lack of systematic knowledge regarding the conceptual domains and constructs of how RCPs have been defined and operationalized in adult mental health inpatient units [26]. This underscores the need to systematically review existing literature and analyse evidence on how RCPs have been defined/Described in the recent literature [27].

## Aims

The aims of the current systematic review were to (1) identify thematic elements within a wide range of definitions used in recent literature to describe the constructs of physical/mechanical restraint, seclusion and chemical restraint in adult mental health inpatient units and (2) synthesize evidence to understand and compare the commonalities and discrepancies in concepts that were incorporated across these definitions. The findings of this review will be crucial in better understanding what is being regarded as RCP and what is being measured and reported. This will help future work in standardizing RCP definitions and their measurement and reporting approaches, thus contributing to a more accurate appraisal of the effect of RCP reduction strategies and to understand the true nature of the actual practice [28].

## Methods

### Design

This paper was a qualitative systematic review. The protocol for this systematic review was registered in the International Prospective Register of Systematic Reviews –PROSPERO (ID: CRD42022335167). We followed the Preferred Reporting Items for Systematic Reviews and Meta-Analyses (PRISMA) guidelines in the overall search strategies and reporting the review findings [29].

### Database search

Before conducting the actual database search, search terms and strategies for this review were developed by all authors through discussion and in consultation with subject librarians from Monash University. A pilot search was done, and minor modifications were made to the search strategy to make it broad enough to adequately address all relevant concepts related to the review outcomes. Six different databases (CINAHL, Medline, Scopus, PsycINFO, Embase and Web of Science) were systematically searched using three main conceptual domains, following the PICO (Population, Intervention and Concept/context) approach [30]. The first concept (Population-**P)** refers to concepts related to psychiatric inpatients and their synonymous terms. The second concept (Intervention-**I)** focuses on concepts related to different forms of RCPs. For this concept, we used different key words and search terms, including physical /mechanical restraint, seclusion and chemical restraint, restriction, immobilization, coercion, sedation and their synonymous terms, to comprehensively address relevant papers. The third searching concept (Context-**C)** represents concepts addressing different mental health/mental illness domains and their synonymous terms.

For this search strategy, authors used different Key-words and Medical Subject Headings (MeSH terms) for each concept to retrieve relevant articles from electronic databases. To create complex search strings and capture variations in word endings and spellings, we utilized Boolean operators within (AND) and between concepts (OR), as well as truncations (e.g., #, $, *, and “) as per the specific requirements of the database (Rao and Moon, 2021). In addition, we manually checked the reference lists of all included articles for the availability of potentially eligible studies. The initial literature search included studies available online from January 1, 2010, to August 15, 2022. Our initial search strategy has been reported in our previous systematic review (Belayneh et al., 2024). We followed the same strategy in the current study. This was however, updated for this paper on October 19, 2023, to include studies that have potentially been published after the initial searching period (Supplementary File 1).

### Study selection

After completing the systematic search of electronic databases and manually reviewing of the reference lists of included studies, all retrieved studies were imported into the Covidence software for screening purposes and to delete duplications First, all authors participated in piloting the eligibility assessment criteria and then (Author 1) did the title and abstract screening. Next, two authors (Author 1 and Author 2) independently completed the full-text review for each study using the same eligibility assessment criteria. A third author (Author 5) participated in resolving conflicts between the two reviewers’ decisions during the full-text review.

This review specifically examined studies conducted in adult mental health inpatient settings, without any geographical limitations. However, the authors have excluded studies conducted in forensic, geriatric, addiction, youth, and adolescent psychiatric inpatient settings due to significant differences in managing individuals with mental health challenges in these contexts. For instance, in forensic settings, patients may be legally prohibited from leaving a designated area as mandated by national law. This directly relates to the definition of restrictive care practice use, which are beyond the capacity of clinicians. The detailed descriptions of the inclusion and exclusion criteria for this systematic review have been presented in Table 1 (Table 1).

**Table 1:** Eligibility assessment criteria used to include or exclude studies to this systematic review

Assessment criteria	Inclusion	Exclusion
Study settings	Mental health inpatient settings	Mental health outpatient departments, Nursing homes, Aged care and Forensic settings
Outcomes	Physical/mechanical restraint, seclusion and/or chemical restraint	Absence of at least one of the three review outcomes
Clinical conditions	Primary psychiatric disorders as per the DSM criteria	Developmental and neurocognitive disorders (such as dementia and delirium), addiction, and other somatic disorders.
Study sample	Adult population	Paediatric or geriatric populations
Publication language	English	Publications other than English languages or those that did not have English language translations
Publication year	From 2010 to 2023	Publications before 2010

For chemical restraint, there were difficulties in determining whether a practice was chemical restraint, or if a medication was administered as a standard treatment care plan. In psychiatry, some medications that are used to treat medical conditions (e.g. anti-psychotics) are also often used for restraint purposes, creating confusions when defining chemical restraints. As a result, we included studies only if it was clearly stated that the primary purpose/s of the medication administration was for restraint purposes. In other words, studies were excluded if the primary reason for medication administration was not mentioned, or the healthcare practitioner’s primary intention was to treat medical symptoms.

### Review outcomes

This review had three main outcomes, including the definitions of physical/mechanical restraint, seclusion, and chemical restraint.

- **Physical/mechanical restraint**: For this outcome, we use “physical/mechanical restraint” to cover restraint performed both by applying human force/Pressure and equipment devices.
- **Seclusion**: The term “seclusion” and other synonymous terminologies such as isolation, confinement, or locked rooms that have been used to describe the concept of seclusion were considered.
- **Chemical restraint**: In the case of chemical restraint, we considered studies that have explicit definitions of chemical restraint or clearly stated that the primary desired outcome from the medication/drug was restraining the person.

### Data extraction

Data extraction for this study was conducted using a custom-developed spreadsheet prepared in Microsoft Excel format. All authors initially developed the data extraction tool and pilot-tested it with a randomly selected sample of eligible articles to check whether all authors agreed on which data should be collected and whether the data extraction tool was adequate to encompass the data required to address the review objectives. After the pilot data extraction, minor modifications were made to the format of the data extraction tool to accommodate the different data classification and presentation options. Four authors extracted data for a randomly selected 15% of the included studies. Author 1 then completed the data extraction for the remaining studies with frequent advisory inputs and feedback from the other co-authors (Authors 3, 4 and 5).

For studies that provided explicit definitions of physical/mechanical restraint, seclusion and or chemical restraint, all verbatim statements describing these definitions were copied from each study and pasted into the spreadsheet for coding purposes. Explicit definitions are straight-forward statements that clearly describe the definitions of restrictive care practices (e.g. Seclusion is “defined as” …or … “is called” …. or … “refers to”…) [31]. However, some other studies did not have explicit definitions of RCPs, but rather they indirectly implied their operational definitions within some sections of the paper [32]. Implicit definitions refer to implied meanings that are indirectly understood from the context without being directly stated, often inferred from context or underlying assumptions [33, 34]. Implicit definitions were identified from different sections of the paper, including descriptions of the outcome data collection approach for physical/mechanical restraint, seclusion and/or chemical restraint, as well as the reporting of results and within the text and labels in tables used to report results regarding their use. For instance, we identified an implicit definition of seclusion from the methodological descriptions of a study (e.g. …*in our settings*,* patients are being locked in a single room with a maximum interval of 15 minutes*,* in some cases when danger to self*…”) [35].

In this review, conducting a risk of bias assessment was not considered relevant as the primary aim of the review was to identify and characterize the definitions of restrictive care practices that have been used in the recently published literature. In other words, we extracted data on how studies have defined restrictive care practices, and the data we extracted would not be affected by the methodological qualities of the included studies.

### Data coding and analysis

In this review, inductive content analysis was used to synthesize evidence and comprehend the commonalities or discrepancies in conceptual boundaries when defining physical/mechanical restraint, seclusion, and chemical restraint across literature [36]. We followed a series of steps to identify, code and categorize individual information elements incorporated within each definition of different forms of RCPs into meaningful units.

In the first stage, authors read and re-read the verbatim descriptions for each definition of physical/mechnaical restraint, seclusion and chemical restraint to gain a comprehensive understanding of the overall message and implications of the concepts incorporated with these definitions. Second, messengers incorporated within each definition were identified to inform the categorization of meaningful units or components. Third, the message segments/meaning units and fine-grained codes were identified to label or tag portions of the data that represent specific concepts. This procedure entails dissecting the data or concepts into finer categories or codes, capturing the intricacies and variations within the broader conceptual domains. Fourth, the coded meanings derived from the fine-grained codes were condensed to generate a smaller number of broader concepts (themes) that could encompass multiple conceptual codes and categories. Finally, individual codes and categories with similar conceptual elements were inductively grouped together into the generated emergent themes. This process involved systematically re-organizing and re-grouping the codes and categories based on their shared characteristics or underlying meanings. By consolidating related codes and categories under the identified concepts or themes, we developed a structured framework that represents the main thematic elements available in the definitions of physical/mechanical restraint, seclusion and chemical restraint [37].

All authors participated in the initial data coding, development and refinement of (sub)categories and themes, and discussed how to refine the terminologies used to express the coding items, categories and themes.

## Results

### Search results

**Fig. 1:**
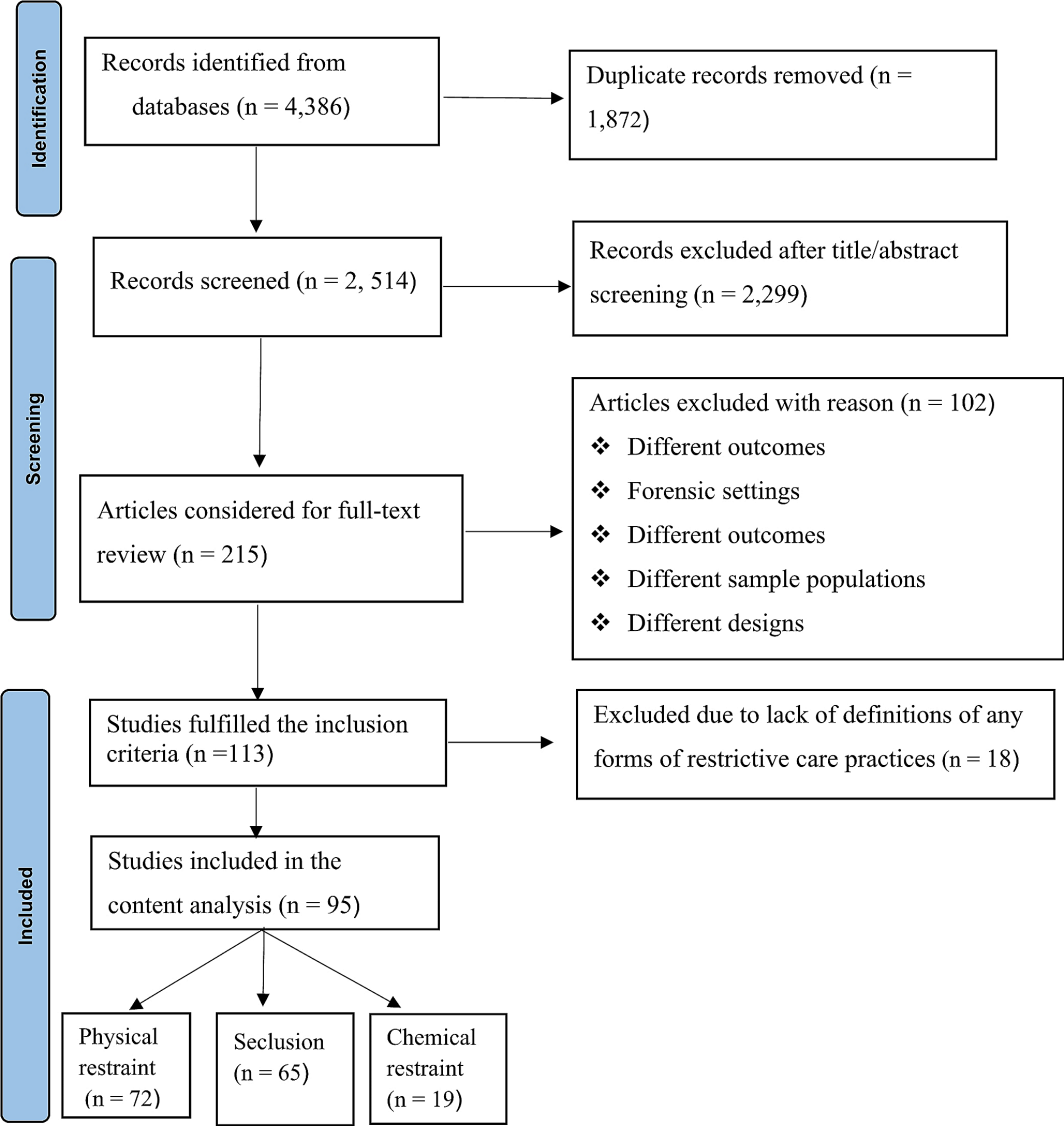
Flow chart of systematic literature search and study selectionusing PRISMA. **Note**: Some studies had definitions for more than one of the review outcomes that the sum of studies included to in the analysis of physical/mechnical restraint, seclusion and chemical restraint is greater than 95

There were 4,386 studies identified from the electronic database search and manual review of reference lists of included studies. Nearly half (*n* = 1,872) were excluded due to duplications, and 2,514 were considered for the title/abstract screening. We excluded 2,299 studies during the title/abstract review, and other 102 studies were excluded during the full-text review. We found 113 studies that fulfilled the inclusion criteria, but 18 were further excluded due to the absence of clear definitions (either explicit or implicit) of physical/mechanical restraint, seclusion or chemical restraint. Finally, 95 studies were included in the content analysis of this study (Fig. 1). The reference lists of all studies included in this review have been submitted as a supplementary file (Supplementary File 2).

### Original characteristics of included studies

From a total of 95 studies that have been included in this review, the majority (*n* = 39) were from Europe, followed by North America (*n* = 21), Asia (*n* = 17), Australia (*n* = 14), Africa (*n* = 2) and South America (*n* = 2). Nearly half (*n* = 42) of the studies only had definitions for one form of RCP, omitting comprehensive coverage of the concept and the availability of different RCP techniques used in inpatient mental health settings. Some studies provided definitions only for physical restraint (*n* = 13), only for seclusion (*n* = 20), and only for chemical restraint (*n* = 4). Conversely, 47 studies had definitions for more than one of the review outcomes [11 studies had definitions for all three review outcomes (physical/mechanical restraint, seclusion and chemical restraint), 34 studies had definitions of physical restraint and seclusion, and four studies had definitions for physical and chemical restraint]. In other words, 72, 65, and 19 studies had definitions for physical/mechanical restraint, seclusion and chemical restraint, respectively, and were included in the content analysis of this review. Compared with physical restraint and seclusion, the definition of chemical restraint was not provided as much in the literature (Table 2). Although most of the reviewed studies provided explicit definitions, there were studies that did not explicitly define their research outcomes. Instead, they indirectly suggested their implicit definitions for physical/mechanical restraint (*n* = 11), seclusion (*n* = 12), and chemical restraint (*n* = 5) in some sections of the paper (Table 2).

**Table 2:** Definitions used in published studies to describe the constructs of physical/mechanical restraint, seclusion and chemical restraint in adult mental health inpatient units, *n* = 95

Author’s name and publication year	Study’s country of origin	Forms of restrictive care practice	Definitions applied
(Anderson et al., 2021)	USA	Physical restraint	Use of physical force or mechanical devices “as a restriction to manage a patient’s behaviour or restrict the patient’s freedom of movement.”
		Seclusion	Involuntary confinement of a patient alone in a room or an isolated environment
(An et al., 2016)	China	Physical restraint	Immobilisation with mechanical devices
(Barnett, 2018)	Malawi	Physical restraint	Holding a patient and restricting their movement using belts or other devices
		Seclusion	The act of involuntarily confining a patient to a room where they are unable to exit
		Chemical restraint	Using involuntary medications to calm or sedate a patient
(Beaglehole et al., 2017)	New Zealand	Seclusion	Initiated by nursing staff as an intervention of last resort for managing a situation of imminent or actual violence.
(Bergk et al., 2010)	German	Physical restraint	The use of belts, handcuffs, and the like, which restricts the patient’s movement or totally prevents the patient from moving
		Seclusion	An involuntary confinement of a person in a room or an area where the person is physically prevented from leaving
(Bilanakis et al., 2010)	Greece	Physical restraint	Use of belts to secure a patient to a bed
		Seclusion	Placing a patient in to an empty, locked room
(Bilanakis et al., 2011)	Greece	Chemical restraint	Emergency intramuscular drug administration for the management of patients’ acute agitation and violent behaviour
(Bowers et al., 2012)	UK	Seclusion	Patients were asked to stay in room or area for period, without the door being locked’.
(Brady et al., 2017)	Australia	Physical restraint	Physical restraint may be used within psychiatric inpatient units in wards and elsewhere to manage behaviour
		Seclusion	Patients were restricted on leaving the ward
(Bullock et al., 2014)	Australia	Seclusion	The last-resort emergency measure to involuntarily control an individual experiencing serious mental health crisis in psychiatric inpatient hospital settings
(Chavulak and Petrakis, 2017)	Australia	Seclusion	The sole confinement of a person to a room or any other enclosed space from which it is not within the control of the person confined to leave”
(Chiba and Subramaney, 2015)	South Africa	Seclusion	Involuntary confinement of an agitated, unstable person alone in a contained, controlled environment for patients who are at a risk of harm to themselves or others
(Chieze et al., 2021)	Switzerland	Physical restraint	Physical or mechanical immobilization aiming to influence a patient’s choice
		Seclusion	Containment of a patient in a closed room that he/she cannot exit freely
(Cole et al., 2020)	German	Physical restraint	Mechanical restriction of a patient’s freedom of movement using special fixation straps
		Seclusion	The supervised isolation of patients in a special isolation room
(Di Lorenzo et al., 2012)	Italy	Physical restraint	All handling, physical, and mechanical methods applied to the patient to reduce his or her freedom of movement or access to his or her own body were defined as physical restraints
		Seclusion	Isolation of the patient in an enclosed space to immobilization of the patient by the staff
(Danielsen et al., 2019)	Denmark	Physical restraint	Restraining a patient to a bed using belts or straps to avoid patients from harm to themselves or others
(De Hert et al., 2010)	Belgium	Physical restraint	Physically restricting movement to confining the limbs on a specially designed bed (that is ‘four-point’ or ‘five-point’ restraint), but it can also mean restraining patients to a chair, limiting arm or leg movement or restraining the whole body with a camisole or a straight jacket
(Dumais et al., 2011)	Canada	Physical restraint	Use of straps, belts or other equipment to restrict movement.
		Seclusion	Temporary isolation of a patient in a purposefully designed room; the room is usually non-stimulating, bare or sparsely decorated, is locked from the outside and generally has a window for observation.
(Duxbury et al., 2019)	UK	Physical restraint	A skilled hands-on method involving trained, designated healthcare professionals” designed to safely immobilize an individual to prevent them from harming themselves, endangering others, or seriously compromising the therapeutic environment.
(El-Abidi et al., 2021)	Spain	Physical restraint	Immobilization of a person through the application of mechanical devices that cannot be easily controlled or removed to prevent free movement of their body
(Feeney et al., 2022)	Ireland	Physical restraint	Safely immobilizing an individual to prevent them from harming themselves or other patients
		Seclusion	Temporary isolation of a patient in a purposefully designed room; the room is usually non-stimulating, bare or sparsely decorated, is locked from the outside and generally has a window for observation.
(Flammer et al., 2022)	German	Physical restraint	The use of physical force for the purpose of preventing the free movement of a resident’s body
		Seclusion	Locking a person in a scarcely furnished room (mostly with only a mattress and toilet) without the presence of staff
(Flammer and Steinert, 2016)	German	Physical restraint	The use of belts to fix a patient to a bed
		Seclusion	Bringing a patient to a locked room from which she or he is unable to leave but in which she or he can move freely
(Flammer et al., 2021)	German	Physical restraint	Encompassing not only belts in beds, but also (undivided) bedrails, movement-restricting blankets, tables attached to a chair, and other devices or staff holding a person for a period of time by force
		Seclusion	Locking a person in a scarcely furnished room without the presence of staff
(Flammer and Steinert, 2015)	German	Physical restraint	use of belts to fix the patient to the bed
		Seclusion	Bringing the patient into a locked room where he or she is alone and able to move freely but unable to leave due to a locked door
(Hotzy et al., 2018)	Switzerland	Physical restraint	Patients are strapped to a bed with mechanical devices (belts)fixed the patient’s arms, legs and torso accompanied by staff during the whole time
		Seclusion	Patients are being locked in a single room with surveillance through a window with a maximum interval of 15 min and, in some cases when danger to self may need immediate response
		Chemical restraint	Coercive medication can be used as an acute intervention, orally or as an intramuscular injection
(Fukasawa et al., 2018)	Japan	Physical restraint	Fixation of a patient by mechanical devices such as clothes or an insulated band to suppress his/her movement
		Seclusion	Isolation of a patient in a locked room, from which the patient is unable to exit by his/her choice, to keep the patient away from other patients
(Georgieva et al., 2012)	Netherlands	Physical restraint	The application of any mechanical device which limited the patient’s movement, physical activity, or normal access to his or her body
		Seclusion	The placement of a patient in a locked room from which free exit is restricted
		Chemical restraint	Rapid tranquillization involved the oral or intramuscular administration of a combination of haloperidol and promethazine, or lorazepam to achieve rapid, short-term behavioural control of any extreme agitation, aggression or potentially violent behaviour that placed the individual and those around them at risk
(Gowda et al., 2018)	India	Physical restraint	At least one or more of the patient’s limbs are fixated by a mechanical appliance or at least one or more of the patient’s limbs is held by staff for treatment purpose or to disengage from harmful behaviour of the individual
		Seclusion	Placement of an individual patient in a locked room against patient’s will to disengage from harmful behaviour displayed by an individual, temporarily restricting contact with the external world and it leads to restriction/loss of patient’s freedom and liberties
		Chemical restraint	Use of forceful injection of psychotropic medication either intramuscularly or intravenously against the patient’s consent/will by staff for treatment purposes or to disengage from harmful behaviour displayed by an individual
(Griffiths et al., 2018)	UK	Seclusion	The supervised confinement of a patient in a room, which may be locked
(Guzman-Parra et al., 2021)	Spain	Physical restraint	The application of mechanical fastening devices to limit physical mobility to prevent damage to the patient, other individuals and/or the physical environment that surrounds them.”
(Guzmán-Parra et al., 2022b)	Spain	Physical restraint	The application of a device (e.g. belts, a vest or a straitjacket) to restrict the person’s movement in emergency situations in order to prevent damage to the user, other people and/or the physical environment that surrounds them
(Guzman-Parra et al., 2015)	Spain	Physical restraint	The application of mechanical fastening devices to limit physical mobility in order to prevent damage to the patient, other people, and/or the physical environment that surrounds them.
(Guzman-Parra et al., 2016)	Spain	Physical restraint	Any manual method or physical or mechanical device, material, or equipment that immobilizes or reduces the ability of a person to move his or her arms, legs, body, or head freely
(Haefner et al., 2021)	USA	Seclusion	Isolating an individual away from others by physically restricting their ability to leave a defined space by locking someone in a defined space or containing them in a specific area by locking access doors or by telling them they are not allowed to move from a defined space and threatening or implying negative consequences if they do
(Hendryx et al., 2010)	USA	Physical restraint	Involuntary restriction of a patient’s freedom of movement, physical activity, or normal access to his or her body
		Seclusion	Involuntary confinement of a patient alone in a designated room where the patient was prevented from leaving
(Hilger et al., 2016)	German	Physical restraint	Restraints were fixed at five points, both arms and both legs and trunk
(Hirose et al., 2021)	Japan	Physical restraint	Restraint with a cloth or band specially made for restraint mainly the hands, wrists, waists, ankles, or a combination
(Huf et al., 2012)	Brazil	Physical restraint	Restraints are with strong cotton bands to both arms and both legs and attached to the bedside to allow some restricted movement in the prone position
		Seclusion	Patients were stayed in a locked room with minimal bedding but bright and airy with good day light though barred windows with no frame or glass open to the nursing station
(Husum et al., 2010)	Norway	Physical restraint	Strapping a patient to a bed with mechanical devices and a bed with belts over the patient’s arms, legs, and torso
		Seclusion	Confining a patient in a single room or separate unit/area inside the ward, accompanied by staff
(Hu et al., 2019)	Australia	Chemical restraint	The administration of medication in an emergency and on an involuntary basis to control the behaviour of a person to prevent the person from harming him/herself or endangering others.
(Jacob et al., 2016)	USA	Physical restraint	Any manual or physical or mechanical device, material, or equipment attached to or adjacent to the resident’s body that the individual cannot remove easily which restricts freedom of movement or normal access to one’s body
		Chemical restraint	Administration of any drug used for discipline or convenience but not required to treat medical symptoms.
(Janssen et al., 2013)	Netherland	Seclusion	Placing a patient in a locked room being alone and able to move around but unable to leave due to a locked door
(Jegede et al., 2017)	USA	Physical restraint	Use of certain apparatuses which restrict a patient’s movement and which the patient is unable to remove. This term may also apply to the use of any apparatus which otherwise is not normally used for this purpose, if the patient is not able to release the mechanism
		Seclusion	Involuntary confinement of a patient alone in a room and physically preventing them from leaving for any period. It could also involve placing the patient in a locked room or in a room with the door held shut that restricts a patient’s freedom or keeps them separated from a group
		Chemical restraint	The use of medications to control behaviours or restrict a patient’s freedom of movement and is not standard treatment or dosage for the patient’s medical or psychiatric condition
(Jayaram et al., 2012)	Maryland	Seclusion	Placing patient in a quiet room with time schedules
(Jury et al., 2019)	New Zealand	Seclusion	Placing a person ‘alone in a room or area, at any time and for any duration, from which they cannot freely exit’
(Knutzen et al., 2013)	Norway	Physical restraint	Use of different types of belts (for restraint in bed or used outside bed for arms and feet only).
		Seclusion	Detention for a short period (up to 2 h) behind locked or closed doors without a staff member present.
		Chemical restraint	Single doses of medications with an antipsychotic or sedative effect that are given by injection or taken orally
(Kuppili et al., 2022)	India	Physical restraint	The application of physical methods to limit the freedom of movement of the patient who is agitated and, therefore, at risk of harming himself or others
		Chemical restraint	Giving injectable medications that decrease agitation and undesirable behaviours by sedating the patient, against the patient’s will.
(Lai et al., 2019)	New Zealand	Seclusion	A consumer is placed alone in a room or area, at any time and for any duration, from which they cannot freely exit’
(Laila et al., 2019)	Indonesia	Physical restraint	Tying hands with rope, use of wood or leg chains to restrict movements
		Seclusion	Isolation/confinement for at least one day
(Larue et al., 2013)	Canada	Physical restraint	Use of equipment (e.g. belts) or holding the patient physically to limit/prevent movement
		Seclusion	Temporarily removes them from a public environment and isolates them in a location that is deemed safe and that they cannot leave at will
(Lee et al., 2010)	Australia	Seclusion	Placing into a secure locked room, is used to contain potentially harmful behaviour when other interventions have been unsuccessful in stabilising or reducing risk of imminent harm
(Leerbeck et al., 2017)	Denmark	Physical restraint	Belt fixation possibly combined with the use of straps or gloves, involuntary sedative drug administration, physical retention, locking of doors, and personal shielding
(Lepping et al., 2016)	Australia	Physical restraint	Use of physical force (by one or more persons) for the purpose of preventing the free movement of a resident’s body when he or she poses an immediate threat of serious harm to self or others’’.
		Seclusion	Placing or leaving of a person in any room alone, at any time, day or night, with the exit door locked or fastened or held in such a way as to prevent the person from leaving
(Lickiewicz et al., 2020)	Poland	Seclusion	Belt fixation possibly combined with the use of straps or gloves, involuntary sedative drug administration, physical retention, locking of doors, and personal shielding),
(Lykke et al., 2019)	Denmark	Physical restraint	Fixation by a mechanical device, which includes immobilization with leather belts
(Mah et al., 2015)	Denmark	Physical restraint	Physical or manual restraint applied by staff to a patient to restrict the patient’s movement
		Seclusion	Any room that confines the patient and prevents the patient from feely exiting
		Chemical restraint	Administered of medications to a patient to achieve an immediate level of control over agitation and threatening, destructive, or assaultive behaviours to prevent harm to self or others excluding the use of psychotropic medication for treatment purposes
(McLeod et al., 2017)	New Zealand	Seclusion	Service user is placed by themselves in an area or room from which they cannot freely exit.
(Miodownik et al., 2019)	Israel	Physical restraint	The use of belts attached to a bed in a special single-bed room in order to restrict the movement of each of the patient’s arms and legs
		Seclusion	Placing the patient in a locked upholstered room
(Nawka et al., 2013)	New Zealand	Physical restraint	Fixing at least one of the patient’s limbs with a mechanical device or being held by a staff member for longer than 15 min
		Seclusion	Involuntary placement of an individual locked in a room alone, which may be set up especially for this purpose
		Chemical restraint	Involving at least three staff members to administer medication against the patient’s will
(Nakamura et al., 2013)	Japan	Chemical restraint	Patients who were given intravenous or intramuscular haloperidol injection on the first day of admission (the haloperidol group) or who were initially treated with oral typical antipsychotics or intramuscular levomepromazinezine injection were excluded
(Noda et al., 2013)	Japan	Physical restraint	The use of restraining straps, belts, or other equipment to restrict movement, OR physically holding an individual, preventing movement.
		Seclusion	Isolation of an individual in a locked room
(Noorthoorn et al., 2016)	Netherlands	Physical restraint	Immobilizing the patient with external mechanical devices or physical force or belts to fix a patient to a bed or chair t on the floor or upright position by staff members
		Seclusion	Bringing the patient into a locked room where he/she is alone and able to move around, but unable to leave the door
(O’Callaghan et al., 2021)	Ireland	Physical restraint	The use of physical force for the purpose of preventing the free movement of a resident’s [patient’s] body when he or she poses an immediate threat of serious harm to self or others
		Seclusion	Placing or leaving of a person in any room alone, at any time, day or night, with the exit door locked or fastened or held in such a way as to prevent the person from leaving’
(Odgaard et al., 2018)	Denmark	Physical restraint	Restraining of a patient to a bed, without the patient’s consent, by using belt around the waist and/or straps around wrists and ankles to restrict movement
(Pérez-Revuelta et al., 2021)	Spain	Physical restraint	The application of physical restraint devices (wristbands, anklets, belts with magnetic closures and restraint bands) to restrict the physical mobility of a patient
(Poloni et al., 2020)	Italy	Physical restraint	Use of any mechanical device that immobilizes or reduces patient’s ability to move
		Seclusion	Limitation of personal freedom to access all areas of the environment
		Chemical restraint	The use of medications in order to obtain sedation
(Prinsloo and Noonan, 2010)	Ireland	Seclusion	Locking a patient alone in a room for protection of the patient and his environment and in order to control problem behaviour and to enable nursing and treatments
(Raboch et al., 2010)	Different European countries	Physical restraint	Fixing at least one of the patient’s limbs with a mechanical device or being held by a staff member for longer than 15 min
		Seclusion	Involuntary placement of an individual locked in a room alone, which may be set up especially for this purpose
(Reitan et al., 2018)	Norway	Physical restraint	Straps on limbs and/or chest binding the patient to a bed, or, in some cases, straps used to minimize movement during walk or short-lasting hold of the patient.
		Chemical restraint	Administering short-acting medications such as benzodiazepines and antipsychotics per os or as injection
(Saeed et al., 2019)	Pakistan	Physical restraint	Any manual method of use of physical/ mechanical devices / material / equipment attached to a body to restrict movements/ freedom.
		Seclusion	The sole confinement of the person at any hour of day or night in a room in which doors and windows are locked
		Chemical restraint	Medications are carefully administered to enable rapid and short-term behavioural control, which puts people at risk of harm to themselves or others, after failure of initial interventions
(Sampogna et al., 2019)	Italy	Physical restraint	Fixation of at least one of the patient’s limbs by a mechanical device or at least one limb being held by staff for longer than 15 min:
		Seclusion	Involuntary placement of an individual alone in a locked room
(Shahpesandy et al., 2015)	UK	Chemical restraint	The administration of medication to calm or sedate an agitated, violent or aggressive patient as quickly and safely
(Shepherd et al., 2015)	UK	Chemical restraint	Administration of psychotropic medications aiming to quickly calm the severely agitated patient, in order to reduce the risk of imminent and serious violence to self or others.
(Silić et al., 2018)	Croatia	Physical restraint	Physical restraint is ordered if patients are considered an imminent danger to themselves, and cannot remain in a locked seclusion room without actively trying to injure themselves
		Seclusion	Isolation of patients is ordered if patients are considered an imminent danger to others but not themselves, and cannot tolerate or remain in a quiet unlocked room
(Smith et al., 2022)	USA	Physical restraint	Orders placed for violent restraints comprising physical holds, mitts, soft restraints, locking cuffs, or neoprene cuffs (invoked for patient behaviours including behaviours, or inability to exhibit safe behaviours)
		Chemical restraint	medication administration record of a non–long-acting parenteral formulation of a first- or second-generation antipsychotic available on the hospital formulary
(Tyrer et al., 2012)	New Zealand	Physical restraint	A manual method, physical or mechanical device, material, or equipment was used to immobilize or reduce the patient’s ability to move their arms, legs, body, or head freely
		Seclusion	Incidents in which the patient was involuntarily confined to a room or area on the hospital unit, which may include an open or a locked door
(Taylor et al., 2012)	Philippines	Physical restraint	A manual method, physical or mechanical device, material, or equipment used to immobilize or reduce the patient’s ability to move their arms, legs, body, or head freely
		Seclusion	An incident when the patient was involuntarily confined to a room or area on the hospital unit, which may include an open or a locked doo
(Terrell et al., 2018)	USA	Physical restraint	Physical restraint is indicated if the patient is at immediate danger of himself or others.
		Seclusion	Service users are kept is a locked, (but open design which includes unbreakable glasses on doors and walls), so that patients cannot leave
(Staggs, 2020)	USA	Physical restraint	Devices (blanket wraps, net restraints) or holds (physical restraint) and the patient’s time in restraints is reported in minutes (if fewer than 60), hours (if fewer than 24), or days
(Pérez-Toribio et al., 2022)	Spain	Physical restraint	Any action or procedure that prevents a person’s free body movement to a position of choice and/or normal access to his/her body by the use of any method that is attached or adjacent to a person’s body and that he/she cannot control or remove easily
(Välimäki et al., 2022)	Hong Kong	Physical restraint	Any action or procedure that prevents a person’s free body movement to a position of choice and/or normal access to his/her body by the use of any method that is attached or adjacent to a person’s body and that he/she cannot control or remove easily
(Verlinde et al., 2017)	Netherlands	Seclusion	Bringing the patient into a locked room where he/she is alone and able to move around, but unable to leave due to a locked door
(Vruwink et al., 2012a)	Netherlands	Seclusion	Locking up a patient in a room designed for this purpose without opportunities to leave.
(Vruwink et al., 2012b)	Netherlands	Seclusion	Locking a patient in a room designed for that purpose with no opportunity to leave on the patient’s own initiative
(Vruwink et al., 2022)	Netherlands	Seclusion	Solitary confinement in a seclusion room without the option of leaving it
(Wu, 2015)	Hong Kong	Physical restraint	The use of mechanical devices including safety vests, magnetic limb holders, magnetic shoulder straps, pelvic holders, magnetic waists and abdominal belts applied to the patient’s wrists, ankles, shoulders, waist and body that restrict freedom of movement or being secured to the r bed or chair”
(Whitecross et al., 2020)	Australia	Physical restraint	Physical restraint interventions are often used to reduce the imminence and severity of risk
		Seclusion	environmental interventions are often used to reduce the imminence and severity of risk
		Chemical restraint	Restrictive/pharmacological/sedation interventions are often used to reduce the imminence and severity of risk, but can traumatise patients
(Zhu et al., 2014)	China	Physical restraint	The use of belts or other devices to fix a patient to a bed
(Mark et al., 2022)	UK	Seclusion	Seclusion is defined as ‘supervised confinement and isolation of a patient, away from other patients, in an area from which the patient is prevented from leaving
(Cole et al., 2023)	Germany	Mechanical restraint	in the form of restricting a patient’s freedom of movement by fixating them to a bed with special straps designed for that purpose
		Seclusion	referring to the supervised isolation of a patient in a designated locked isolation room where they are allowed to move freely but are not able to leave the room
(Flemmerer et al., 2023)	German	Physical restraint	defined as restraining the patient to a bed with the use of special restraint belts
(Linkhorst et al., 2022)	Demark	Mechanical restraint	Use of gloves/straps, use of belt to fixate patients to bed, retention and force to hold eventually further restraining movements of hands and feet by using straps and further restraining use of fingers by using gloves
		Seclusion	locking of doors at wards (not to patient rooms)
(Guzmán-Parra et al., 2022a)	Spain	Physical restraint	Mechanical restraint is defined as the application of a device (e.g. belts, a vest or a straitjacket) to restrict the person’s movement in emergency situations in order to prevent damage to the user, other people and/or the physical environment that surrounds them
(De Cuyper et al., 2023)	Belgium	Seclusion	The stay of the service user in a specially provided individual seclusion room, or another individual room which the service user cannot leave independently
		Physical restraint	Restraint by means of mechanical devices attached to or immobilized by one or more staff-member in the immediate vicinity of the service user, which cannot be removed independently by the service use

### Definitions of physical restraint, seclusion and chemical restraint

This review identified a wide range of definitions that have been used in recently published literature to describe the constructs of physical/mechanical restraint, seclusion, and chemical restraint. Across the reviewed studies, there was no a universally accepted uniform definition for any form of RCP techniques addressed in this review. The terminologies and conceptual boundaries used to describe different RCPs were inconsistent across studies. For example, some studies used the term “physical restraint” to refer to restraint performed by applying human force/pressure (hand-on immobilization) and “mechanical restraint” to describe the use of equipment devices on the person’s body. Several other studies used the term “physical restraint” to include both type of restraints performed either using either equipment devices or human force/pressure. In this review, we prefered to use “physical/mechanical restraint” to cover restraint performed by both human force/pressure and equipment devices.

The scopes of conceptual boundaries incorporated within each definition were highly inconsistent and varied significantly. The criteria for the inclusion or exclusion of specific intervention practices within the definitions of RCPs have also varied across studies. Some studies used broader definitions that encompassed several forms of RCP techniques and actions, while others used strict definitions and considered only one or two practices to be included in their operational definitions. For example, a study conducted by Di Lorenzo, Baraldi et al. [38] used a broader definition of physical restraint that encompasses several practices: “*All handling*,* physical*,* and mechanical methods applied to the patient in order to reduce his or her freedom of movement or access to his or her own body.*” This definition considers intervention practices and actions that have the effect of reducing the patient’s freedom and access, regardless of the techniques or methods of restraints used, including use of mechanical devices and restraints performed using human force/pressure. On the other hand, a study conducted by Flammer, Eisele et al. [39] defined physical restraint with a very strict and narrow scope: “*Staff holding a person for a period of time by force.*” This definition is limited to a specific method of physical restraint involving staff physically holding a patient, while excluding other forms of restraint practices that can be performed using mechanical devices.

Most of the definitions of chemical restraint had ambiguous explanations that make it unclear to determine whether they are describing restrictive care practices or not. Some definitions lack clear distinction between the concepts of chemical restraint and involuntary administration of standard therapeutic medications. This aspect could be important because some psychotropic medications can be administered both for the purpose of restraint and to treat medical symptoms.

**Common themes generated from definitions: Results of Content Analysis**.

**Fig. 2:**
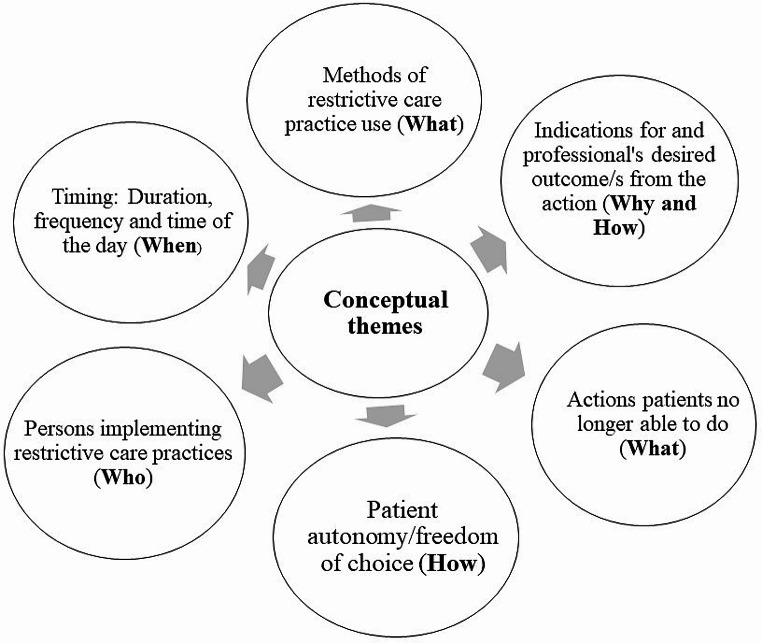
Emergent conceptual themes identified from definitions used by published studies to describe the constructs of physical/mechanical restraint, seclusion and chemical restraint in adult mental health inaptients

There were a wide range of information elements and conceptual boundaries that have been incorporated within the different definitions of physical/mechanical restraint, seclusion and chemical restraint. We identified six emergent concepts (themes) from the data extracted from textual descriptions that studies used to define physical/mechanical restraint, seclusion and chemical restraint (Fig. 2).

None of the definitions included information elements or codes related to all six themes identified in this review. The codes and categories encompassed across these themes were not consistently applicable for the definition of physical/mechanical restraint, seclusion and chemical restraint. Some conceptual elements were specifically identified from the definitions of physical/mechanical restraint, but could not be found within the definitions of seclusion or chemical restraint (Table 3). This resulted in a highly inconsistent number and frequency of codes and categories included within each theme for the definitions of physical/mechanical restraint, seclusion and chemical restraint. The overall codes and categories of concepts identified from the applied definitions of RCPs have been provided in Table 3 (Table 3).

**Table 3:** Codes, categories and (sub) categories of concepts that have been encompassed with in the emergent themes identified from the definitions of physical/mechanical restraint, seclusion and chemical restraint, n = number of definitions that have information elements related to a specific code or category

Themes	Sub-themes and codes		
	Physical restraint	Seclusion	Chemical restraint
**Theme 1: Methods of restrictive care practice use**	**Restraint techniques and material** -Belts (*n* = 23) -Physical force (*n* = 21) -Stramps (*n* = 8) -Straight jacket (*n* = 4) -Bands (*n* = 4) -Blankets (*n* = 4) -Camisoles (*n* = 2) -Nets (*n* = 2) -Vests (*n* = 2) -Cloth (*n* = 2) -Robes (*n* = 2) **Body parts where restraint is being applied** -Arms (*n* = 14) -Legs (*n* = 11) -Both legs and arms (*n* = 2) - Unspecified body part (*n* = 4) -Wrist (*n* = 3) -Waist (*n* = 3) -Torso (*n* = 3) -Ankle (*n* = 3) -The whole body (*n* = 2) -Abdomen 9n = 2) -Shoulder (*n* = 1) **Attachment of devices adjacent to the person’s body** **-**Any equipment (*n* = 13) -Beds (*n* = 7) -Tables (*n* = 4) -Chairs (*n* = 3)	**Area/place of seclusion** -Rooms unspecified (*n* = 29) -Controlled areas/space (*n* = 11) -Purposefully designed rooms (*n* = 7) -Sparsely furnished Rooms (*n* = 5) -Units in hospitals/wards (*n* = 4) -Empty/quiet rooms (*n* = 4) -Fasten/held rooms (*n* = 3) **Availability of other people with the person during seclusion episodes** -Alone (*n* = 26) -Supervised by staff (*n* = 4) -Without staff presence (*n* = 3) -Accompanied by staff or another person (*n* = 1) **Status of door locking** -Locked doors (*n* = 34) -Locked or unlocked doors (*n* = 2) -Unlocked doors (*n* = 1) -Intermittently locked doors (*n* = 2) -Locking the wider area (e.g. main ward) while patient’s room is left open (*n* = 2)	**Medication class/ categories** -Short acting/emergency drugs (*n* = 6) -Any medication (*n* = 6) -Anti-psychotics (*n* = 3) -Not medications (or in a dosage) used to treat medical conditions (*n* = 3) -Psychotropics (*n* = 1) -Benzodiazepines (*n* = 1) -Tranquilizers (*n* = 1) -Corrosive drugs (*n* = 1) **Routes of medication administration** -Injection (*n* = 4) -Intramuscular (*n* = 3) -Oral (*n* = 3) -Injection or oral (*n* = 2) -Intravenous (*n* = 1)
**Theme 2: Indications for and professional’s desired outcome from the action (restrictive care practice use)**	**Violence /risk of harm** -Harm to others (13) Staff (*n* = 2) Other patients (*n* = 3) Others unspecified (*n* = 10) -Self harm (*n* = 9) -Harm to the Physical environment (*n* = 7) Harm to unspecified targets (*n* = 3 -Managing behaviour (*n* = 6) -Last resort option (*n* = 4) -Patient safety (*n* = 3) -Not for clinical observation or medication administration purpose (*n* = 3)	**Violence /risk of harm** **-**Harm to unspecified targets (*n* = 6) -Others (7) Staff (*n* = 3) Other patients (*n* = 2) Others unspecified (*n* = 3) -Self harm (*n* = 3) -Physical environment (*n* = 2) -Managing behaviour (*n* = 6) -Last resort option (*n* = 3) -When the person cannot remain in unlocked room (*n* = 2) Patient safety (*n* = 2) Not by patient request (*n* = 1)	**Violence/risk of harm** **-**Harm to unspecified targets (*n* = 6) -Self harm (*n* = 4) -harm to others (5) Other patients (*n* = 3) Others unspecified (*n* = 2) -Harm to the physical environment (*n* = 1) -Sedation (*n* = 8) -Managing behaviour (*n* = 7 -Patient safety (*n* = 2) -Last resort option (*n* = 1)
**Theme 3: What the person can no longer do during restrictive care practice episodes**	-Movement (*n* = 41) Whole person movement (*n* = 35) Moving of body parts (*n* = 6) -Freedom/choice (*n* = 7) -Access body parts (*n* = 5) -Physical activity (*n* = 3)	-Exit/leaving a room or defined area (*n* = 28) -Freedom/choice (*n* = 8) -Public access/contact with others (*n* = 7) -Not movement around the seclusion area (*n* = 1)	-Movement (*n* = 2)
**Theme 4: Patient Autonomy/Freedom of choice**	**Patient’s will/consent** -Against the persons’ consent/ involuntary (*n* = 12) -Without considering consent (*n* = 3) **Patient capacity** -Inability to easily control the restraint (*n* = 6) -Inability to remove the restraint (*n* = 3)	**Patient’s will/consent** - Against the persons’ consent/ involuntary (*n* = 11) - without considering consent (*n* = 3) -Based on patient request (*n* = 2)	**Patient’s will/consent** Against the persons’ consent/ involuntary (*n* = 6)
**Theme 5: Timing: Duration, Frequency, or Time of the day when the action takes place**	-Duration (*n* = 9) -Time of the day (*n* = 3)	-Duration (*n* = 13) -Time of the day (*n* = 9) -Frequency of episodes per 24 h (*n* = 3)	-Frequency of dosage (*n* = 3)
**Theme 6: Persons implementing the action (restrictive care practice)**	-Staff (= 9)	-Staff (*n* = 4)	-Staff (*n* = 9)

#### Theme 1: Methods of restrictive care practice use

This theme describes the details related to the methods of how RCPs have been applied in the clinical settings. In this theme, the definitions of physical/mechanical restraint had three categories: devices/materials used to apply restraint, body parts where restraints are being applied, and attachment of devices/objects adjacent to a person’s body. Most of the definitions included descriptions for the conceptual category of materials or methods used to apply physical/mechanical restraint. Some studies explicitly mentioned specific types and/or numbers of equipment devices or materials used to impose restraint (e.g., *belts*,* hand holding*,* straps*,* cuffs*,* nets*,* mitts*,* robes*,* and camisoles*), while others had general explanations like “*use of equipment devices*” without clearly identifying which equipment devices can be considered restraint. More than one device or restraint method was mentioned in several studies (e.g., *the application of mechanical devices like belts*,* chins*,* straps*,* or human force*), while a few studies mentioned only one type of restraint material in their definitions. Similarly, some definitions considered concepts regarding the number of restraint points applied on the person’s body where restraint episodes are applied at a time (e.g., *fixing one limb*,* fixing both arms and legs*,* three-point restraint*,* restraining the whole body*) for a practice to be defined as physical/mechanical restraint. The use of weighted items (e.g. *chairs*,* bed and tables)* attached adjacent to the person’s body was also mentioned within some definitions. However, there were inconsistencies regarding the type and number of devices that should be fixed to the person’s body parts for restraint purposes (Table 3**)**.

In the definition of seclusion, this theme also encompasses three different conceptual categories: place/area of seclusion, availability of other people with the person during seclusion episoides in the isolation area/room and the status of door locking. Like the definitions of physical/mechanical restraint, some studies described specific methods or places of seclusion such as *quiet places*,* controlled environments*,* purposefully designed rooms*,* units in hospitals/wards*,* and scarcely furnished rooms* to define seclusion. In contrast to this, others had general statements such as *keeping a person in isolated areas or keeping a person in some place.* Regarding the availability of other people with the person who is under seclusion, most of the definitions agreed with the absence of other people during seclusion, and seclusion was often defined as “*keeping of the patient alone by physically isolating the person from contact with others*”. However, three definitions considered frequent supervision of a person underselling by staff or the presence of other persons (*staff*,* other patients*,* or family caregivers*) around the seclusion area. Over half of the definitions of seclusion stated the necessity of door locking to consider actions as seclusion episodes, while only two studies considered unlocked doors in their applied definitions of seclusion (Table 3**)**. Overall, the controversies in the terminologies and conceptual boundaries of seclusion indicate the variations in the conceptualization and implementation of seclusion in the clinical practice. For example, some units use the term “quiet room” or “isolation room” to designate a room that allows people to voluntarily relax when feeling agitated or stressed [40, 41], while some studies considered this as seclusion episode [42, 43].

For chemical restraint, two categories (medication class/family and routes of administration) were included in the methods theme. Most of the chemical restraint definitions did not mention the names of specific medications used for chemical restraint; instead, they incorporated general terms describing medication categories/classes (*short-acting/emergency medications*,* psychotropics*,* sedatives*,* anti-psychotics*,* tranquilizers*,* benzodiazepines*,* corrosive drugs*,* and medications with unspecified categories)*. Three definitions of chemical restraint considered more than one drug category option (e.g., *use of anti-psychotics or sedatives*) to be used for chemical restraint purposes. Some definitions excluded specific medications or dosage levels that are used as standard therapeutic medication to treat medical conditions from their definition of chemical restraint [44–46]. Regarding the routes of medication administration, some definitions mentioned specific routes of administration like *oral*,* intramuscular*,* or intravenous*, while others included vague explanations such as “*use of injections*” (Table 3).

#### Theme 2: Indications for and professionals’ desired outcome from the action

This theme encompasses two interrelated concepts. The first one addresses information describing characteristics or conditions observed in patients’ behaviours (e.g., *aggression, self-harm, suicide*) that may lead staff to decide to apply RCPs. The second part is related to the primary intention and professionals’ desired outcomes anticipated from the action/s of RCPs, such as *safety*,* minimizing the risk of harm/violence*,* or preventing the person from unauthorized leave from wards/hospitals or absconding*. During data analysis, we could not exclusively separate these concepts from the data captured, so we have presented the two issues as a single theme. For example, the concept of patient characteristics or behaviour that indicates the necessity of restrictive care practice use conceptually relates to the professional’s desired outcome anticipated from that specific practice.

The sub-categories identified in this theme were: (1) risk of harm or violence, (2) patient safety, (3) managing behaviour and (4) failure of initial less restrictive interventions (restrictive practice as a last resort option). However, the codes or meaning units encompassed across these sub-categories varied for each form of RCP. For example, for the first category (risk of harm or violence), there were variations in the targets of violence or harm mentioned across definitions. These codes included harm towards self, risk of harm to others (other patients, staff, or others not specified) and risk of harm to the physical environment. However, some definitions did not specify the targets of violence *(e.g. to prevent a perceived danger)*, and we have coded them separately as *unspecified target.* The definition of seclusion had additional codes included in this theme, with the inability of the patient to remain in an unlocked room being considered an indication to initiate seclusion episodes. This theme also included codes for concepts used to exclude specific intervention practices from the classification of seclusion episodes. For some definitions, practices and actions were not defined as seclusion if the primary reason for patient isolation was for clinical observation or if it had been initially requested by the patient. In this theme, several studies mentioned “patient *sedation*” to be considered as one of the professional’s desired outcomes expected from the action to define chemical restraint, and this concept was coded separately only for the definition of chemical restraint (Table 3).

#### Theme 3: What patients could no longer do during the RCP episodes

This theme includes conceptual elements that indicate the restrictiveness or severity of the action to limit the patient’s ability during the implementation of RCP episodes. Most definitions described concepts related to the restrictions on patients’ abilities and activities that individuals could no longer do during the implementation of RCPs. Some definitions had descriptions related to different specific tasks which patients could no longer do during RCP episodes, while they were routinely doing them before the initiation of RCP episodes.

Restrictions on a person’s *movement*,* freedom*,* or choice*,* as well as limiting the ability to move body parts*,* access one’s own body parts*,* engage in physical activities*,* interact with others in public and leave/exit from a designated place or room* were identified as codes within this theme. However, these codes were not consistently identified across the definitions of physical restraint, seclusion and chemical restraint. The code “*movement restriction*” was the only one that have consistently identified in the definitions of physical restraint, seclusion and chemical restraint. However, the degree of movement restriction varied across definitions ranging from completely preventing the whole person’s movement to restricting the movement of specific body parts or limiting an individual’s movement within a defined space/area. The codes related to the patient’s ability to *access their own body parts*,* restrictions from engaging in physical activity and movement of body parts* were only applicable to the definition of physical restraint. On the other hand, *lack of access to the public or contact with others and inability to leave or exit a designated area, space or room* were identified only from the definitions of seclusion. Similarly, the code of *reducing patients’ freedom or choice* was a common code for both the definitions of physical restraint and seclusion, but it was not identified from the definition of chemical restraint.

The inconsistencies in the levels of restriction and severity of the action in reducing a patient’s ability during the use of restrictive practice episodes was not only existed between different forms of RCPs, but also found within the definitions of the same practice. For example, most of the definitions of seclusion restrict the person’s movement during RCP episodes. On the other hand, two definitions allow movement of the person within the seclusion area, while restricting the ability to exit/leave from the room or area by their own initiation. without permission from staff or other authorities (Table 3).

#### Theme 4: Time frame: Duration, Frequency and Time of the day when the action of RCP episodes are implemented

This theme includes information related to the time frame, including duration (how long), frequency (how often) and time of the day (when) RCP episodes have been implemented. Only some of the studies considered a time frame for an episode to be classified as a form of restrictive practice within their applied definitions. Codes for “*duration*” and “*time of the day*” were applied only to the definitions of physical restraint and seclusion, but were not found in the descriptions of chemical restraint definitions. In addition, we identified studies that used vague, subjective descriptions such as “*for a brief/transit time*,” “*for some period of time*,” or “*sometime”* to characterize the duration of the action in their definitions of RCP use [45]. Only a few definitions specified an absolute minimum and/or maximum time period (e.g. for more than 15 min [36] or days [42]). However, the minimum duration considered for a single episode to be defined as RCP was variable across studies. Regarding “the time of the day,” only one study described that a practice is defined as restrictive regardless of the time of the day (either day or night) the incident takes place. In this theme, there was only one single code identified for the definition of chemical restraint where “frequency of medication administration” was described as a minimum rate of prescription across definitions (e.g., single dose or as a prescription given on a required basis) (Table 3).

#### Theme 5: Patient autonomy/freedom during the application of RCP episodes

This theme includes concepts that described the extent of a patient’s autonomy to participate in and make decisions about the application of RCPs to themselves. It includes the presence or absence of informed consent, as well as the ability and freedom of patients to accept or refuse orders for RCP use and to easily control or remove these actions of restrictive care practices on their own initiative when they wish. Within this theme, two categories were generated: (1) patient’s will/consent and (2) patient’s ability to easily control or remove restrictive care practices that have been applied to them. Codes related to patient consent were incorporated within most of the definitions of physical restraint, seclusion and chemical restraint. These definitions showed agreement in the involuntary application of RCPs against the patient’s consent or without attempting to secure patient consent. Codes related to a person’s ability to easily control and/or remove such practices were identified only from the definitions of physical/mechanical restraint, but this concept was not mentioned in the definitions of seclusion and chemical restraint. However, all these definitions consistently stated that the person would not be able to easily control or remove RCP actions on their own initiative. Across the studies that were reviewed, none of the definitions mentioned the possibility of obtaining informed consent from family members or other caregivers on behalf of the service user (patient) [47]. However, this is a common practice in mental health settings, especially for individuals with severe mental health challenges or individuals with cognitive impairments that may affect their ability/competency to make decisions independently [48] (Table 3).

#### Theme 6: Persons implementing the action of RCP episodes

This theme describes concepts regarding personnel and professionals implementing RCPs to the patients. Most of the definitions did not explicitly describe information related to this theme. Moreover, all the definitions that had conceptual elements related to this theme consistently considered “staff” as an authorized personnel who apply these practices. These codes were more frequently mentioned in the definitions of physical restraint and chemical restraint than in seclusion (Table 3). However, the reviewed definitions had limitations in clarifying whether a practice or an action is considered as restrictive care practice or not if the person applying it was a security person (guard) or a family/informal caregiver. In the actual clinical practices, it is a common practice that security persons, such as guards and police involve in the application of restrictive care practices. However, none of the reviewed definitions considered this. Moreover, the types of professionals were not specifically identified in any of the reviewed definitions of physical/mechanical restraint, seclusion and chemical restraint.

## Discussion

This systematic review has identified a diverse range of ways and approaches that recently published literature has used to describe the constructs of physical/mechanical restraint, seclusion, and chemical restraint in adult mental health inpatient units. Specific information elements and conceptual boundaries that have been incorporated across these definitions were highly inconsistent. Thus, the discrepancies in how we define and interpret the concepts of restrictive care practices indicate the overall complexity in understanding and managing these practices in mental health sector, which in turn leads to subjectivities in clinical practices. Discrepancies in defining different forms of RCPs challenge international efforts aiming to minimize the use of restrictive care practices [49]. A common understanding of of RCPs could be crucial for ensuring reliable and valid [49] measurements, as well as for ongoing monitoring and evaluation of the effectiveness of RCP reduction strategies and policy practices.

This review also noted discrepancies across definitions of physical/mechanical restraint, seclusion and chemical restraint particularly in the criteria for determining whether a specific practice or action should be included or excluded from the classification of RCPs according to the applied definitions. Some definitions encompassed a wide range of practices that are considered under the umbrella term of RCP, while others had a narrower focus and excluded certain interventions that are perceived as less restrictive and/or not intrusive. For example, let’s compare two definitions of seclusion: [definition one: placing a person “alone” in a room or area, at any time and for any duration, from which they cannot freely exit; and definition two: confining a patient in a single room or in a separate unit or area inside the ward, “*alone*” or “*accompanied by staff*”]. While both definitions describe the confinement of a person in a single room, the presence or absence of staff during the seclusion period is a crucial distinction in these definitions. The first definition emphasizes the aspect of isolation where a person is “*alone*” and a practice may not be considered a seclusion episode if a staff member or other people are present with the person, but the second definition considers both scenarios as seclusion. Such discrepancies can have implications for how RCPs are implemented, monitored, and evaluated in different clinical settings and policy practices [50], which could lead researchers and clinicians to apply their own subjective interpretations when recording and/or reporting RCP incidents in thier actual workplace [50].

In this review, identifying and categorizing concepts incorporated across the definitions of physical/mechanical restraint, seclusion and chemical restraint were challenging. This stemmed from the absence of clear and explicit definitions of RCPS between studies. Several studies offered implicit definitions through statements containing indirectly suggested implied meanings [34], which were not directly articulated. Implicit definitions encountered in the review were often vague, leading rooms to subjectivity in capturing the actual message applied by these definitions [33]. However, the problems related to the issue of clarity was not actually limited to implicit definitions. There were also several vague explanations across explicit definitions. For example, one of the included studies explicitly defined physical restraint as “*staff holding a person for a period of time by force*” [51]. In the context of this definition, the phrase “*a period of time*” lacks clarity about the absolute minimum and/or maximum duration of the action to be considered as restraint. This creates uncertainty about whether a specific practice should be considered restrictive or not when using this definition as a criterion for classifying RCPs for research purposes or in clinical practices. Only some studies defined physical restraint using maximum and minimum absolute time, but the total duration of time considered for a single episode of RCPs is variable across definitions, ranging from minutes to days [52, 53]. The above definition also seems to narrow down and limit the concept of physical restraint to actions performed specifically by staff members. This limitation excludes the possibility of the involvement of other individuals, such as security personnel (guards), police, family members, or other caregivers in applying RCPs that would otherwise meet the criteria for physical restraint [54, 55]. Such exclusions and narrower scopes in defining RCPs could impede the deeper understanding of by underestimating the actual prevalence and impact of the reduction strategies in reducing these practices in different settings [56].

The primary reason contributing to the observed variations and uncertainties in the conceptualization of RCPs is the lack of standardized definitions and frameworks guiding consistent classification taxonomies for different RCP techniques [57]. Due to the lack of consistent definitions and common frameworks for RCPs at a broader level, healthcare systems and facilities often use different terminologies and create their own subjective definitions to suit their options of interest within their local policies and regulatory requirements [19]. The diversity and inconsistency of terminologies used to describe the concepts of RCP use can be attributed to the complexity in conceptualization and sensitive nature of the issues [58], reflecting the ethical and legal challenges inherent to the clinical practices [17]. The interpretation and understanding of RCPs are further complicated by the culturally bounded perspectives towards mental health issues in general and RCPs in particular [59]. As a consequence, professionals within different organizations may rely on their subjective interpretations and their cultural attributes when interpreting and utilizing RCPs [60]. Some might emphasize the potential benefits of these intervention practices for patient safety and crisis management, while others may underscore its clinical benefits, instead arguing about the legal and ethical issues surrounding patient autonomy and dignity [61].

In addition to the absence of common definitions, the political and legislative contexts of different countries could have a significant impact on how restrictive practices are defined, considered, and monitored. The political priorities of a nation or local institutions can influence the level of oversight and the initiatives taken to reduce these practices. Countries that prioritize human rights tend to have strict regulations and robust monitoring systems in place. This helps minimize the use of restrictive practices by establishing clear criteria and safeguards to define which actions are restrictive and which are not. On the other hand, countries with less comprehensive legislation face challenges in ensuring consistent managing these practices. Therefore, it is also crucial to consider political and legislative contexts alongside common definitions to effectively apply these definitions while monitoring the use of RCPs. The subjectivity surrounding the conceptualization of RCPs can also affect the documentation and reporting practices of RCP incidents, limiting the generalizability of research findings to represent the true nature of the actual practices in the field [62]. This calls for a joint strategy and learning process to ensure such practices are appropriately and consistently regulated and reported across different institutions [14]. This study systematically reviewed the different ways in which RCPs in mental health settings have been defined, setting the scene for an appeal for greater consistency in research and practice to enable valid and relabel comparisons of international data collected from different regions and jurisdictions, and to support improvements in clinical care. Consistent definitions of RCPs support efforts to harmonize regulations and guidelines, ensuring that individuals receive equitable and high-quality care regardless of geographic locations and differences in behaviour or symptom presentations [17]. While achieving a consensus definition can indeed be complex and challenging task, requiring a more comprehensive evaluation of the current definitions of RCP, researchers and clinicians are advised to critically assess and prioritize existing definitions to consider for describing such practices. This process allows them to tailor the definitions to suit their specific context and objectives, facilitating the adoption of a more comprehensive approach [63]. Such an approach can also serve as a solid foundation for future research works aiming at establishing common ground for a shared understanding of RCPs [64].

The majority of the conceptual categories and codes identified from the definitions of physical/mechanical restraint in this review showed good agreement with another similar systematic review conducted in long-term care [65]. However, there were discrepancies in the information elements presented for chemical restraint definitions in the current review compared to the long-term care paper. Specifically, the long-term care paper codes related to routes of medication administration, patient consent, individuals implementing chemical restraints, and exclusions of specific medications or dosage levels from the definition of chemical restraint were not reported. Differences in the utilization of psychotropic medications between mental health and long-term care settings could in fact contribute to variations in how chemical restraint is conceptualized and implemented [66, 67]. In mental health settings, psychotropic drugs are more frequently used both as a standard treatment plan or for restraint purposes than in long-term care settings due to the differences in the patient conditions between the to settings [68]. This may drive the need for more detailed explanations of chemical restraints to distinguish it from traditional pharmacological interventions used to treat medical symptoms in mental health sectors [69]. Moreover, the sparsity of studies available for inclusion in long-term care settings may have constrained the depth and breadth of information available for analysis of chemical restraint definitions in the long-term care paper. For the definitions of seclusion, conceptual domains identified in the current review showed similarities with another systematic literature review conducted by Mason [70]. However, the current review identified additional conceptual elements related  *patient behaviour or characteristics indicating the need to initiate seclusion and persons implementing action* that were not reported in Mason’s study. This is possibly due to the time difference between our review and Mason’s review (published in 1999). The increased attention and emphasis on managing and understanding RCP over time may change approaches in the way how RCPs have been defined in recent studies [71].

## Limitations

The main limitation of this systematic review is its exclusive focus on the definitions that have been used by published literature to describe the constructs of physical/mechanical restraint, seclusion and chemical restraint inadult inpatient mental health units. This review did not examine how different mental health stakeholders are actually interpreting and defining RCP incidents at the actual clinical practice. Therefore, it is crucial for future studies to examine evidence to clearly understand variations and/or similarities in how clinicians, researchers and policymakers define RCPs in their actual workplace. This can provide inputs to move forward for the development of tailored and culturally contextualized definitions that can reflect the unique needs of different communities, maintaining consistent classification of incidents across regions [72]. The other limitation of this review could be the inclusion of a small number of studies for the definition of chemical restraint. This is because studies did not provide clear definitions of chemical restraint as such in the literature. It was also difficult to identify whether the professional’s desired outcome from practice was to manage medical symptoms or for restraint purposes. As a result, this review included only studies that either directly referred to the term “chemical restraint” or implied that the primary intention of medication/s administration was for restraint purposes. The third limitation of this review was the exclusion of studies published in non-English languages. These studies could potentially provide unique perspectives and insights to comprehensively understand concepts associated with the constructs of RCPs in mental health settings. A fourth limitation of this study is that the review authors have applied their judgment when extracting data from non-specific or vague texts indicating implied definitions, which may potentially affect the review outcome. However, there were only a small number of studies (< 10%) that required this judgment.

## Implications and future directions

The current review identified significant gaps in existing literature related to the lack of clarity and uniformity in describing and categorizing different actions in the family of RCPs. The absence of standardized definitions for restrictive care practices in adult mental health inpatient settings has profound implications. For example, inconsistent definitions hinder the consistent application, measurement and reporting of these practices across hospitals and among professionals, which in turn hampers the quality of care. The legal and ethical challenges arising from varying definitions further complicate the justification and implementation of these practices. Moreover, unreliable data resulting from inconsistent definitions of measurement outcome that do not reflect the naturally occurring practices would potentially create errors and measurement biases. This negatively affect evidence-based practices, eroding the therapeutic relationship between professionals and service users. These together hinder the person’s recover [65] and will lead to staff burnout and dissatisfaction. This emphasizes the urgent need to develop more precise and consistent guidelines for the terminologies and descriptions used to consistently define different forms of RCPs across different settings and hospitals [73].

The findings of this review provided insight in to understanding various definitions and interpretations of RCPs used in recent studies. This would lay the groundwork for consensus-building and the development of coherent guidelines and practices to consistently define and manage RCP use in mental health sectors [64, 74]. By cross describing the conceptual themes generated in the review, researchers and clinicians can gain a deeper understanding of both the shared elements and unique variations in the key components that characterize the concepts of RCPs [75]. This allows to consider various viewpoints and interpretations during the consensus -building process [25,76]. During the establishment of consensus definition, taking local contexts into consideration is a pragmatic solution for designing and implementing efficient strategies to define and manage restrictive care practices [16, 17]. Collaboration among mental health organizations, regulatory bodies, and service users is essential in order to pursue opportunities, offer comprehensive training, and advocate for policy reforms [11, 12]. These efforts would significantly bolster the safety, quality, and uniformity of care in mental health facilities [13].

## Conclusions

The identified gaps in the existing literature concerning the definitions of RCPs call for the development of more precise and consistent guidelines. The lack of consensus or unified definitions of restrictive care practices within the existing literature indicates a divergence in interpretations and variations in actual practices. Without common definitions, clinicians often disagree on determining which practices are restrictive and should be documented in restrictive care practice reporting systems and which are not, challenging efforts to reduce the use of restrictive care practices. It is crucial to endorse universally accepted, uniform definitions, and classification taxonomies for various forms of restrictive care practices. Clear and consistent definitions allow for accurately measuring actual practices and making comparisons across studies and data collected from the international level. This enables a better understanding of the true nature of restrictive care practice use across different settings and policy practices. This calls for collaboration among many stakeholders to design protocols and develop policies that can guide clinicians in consistently defining, managing, and reporting restrictive care practices to improve the quality of care for people receiving mental health services.

## Electronic supplementary material

Below is the link to the electronic supplementary material.

Supplementary Material 1

Supplementary Material 2

## Data Availability

No datasets were generated or analysed during the current study.

## References

[1] Lickiewicz J, Adamczyk N, Hughes PP, Jagielski P, Stawarz B, Makara-Studzińska M (2021) Reducing aggression in psychiatric wards using Safewards—A Polish study. Perspect Psychiatr Care 57(1) 10.1111/ppc.12523 32363654

[2] Kawai Y, Hamamoto M, Miura A, Yamaguchi M, Masuda Y, Iwata M, Kanbe M, Ikematsu Y Prevalence of and factors associated with physical restraint use in the intensive care unit: a multicenter prospective observational study in Japan. Intern Emerg Med 2021:1–6 10.1007/s11739-021-02737-5 33852145

[3] Wilson C, Rouse L, Rae S, Kar Ray M (2017) Is restraint a ‘necessary evil’in mental health care? Mental health inpatients’ and staff members’ experience of physical restraint. Int J Ment Health Nurs 26(5):500–512 28960742 10.1111/inm.12382

[4] Chieze M, Hurst S, Kaiser S, Sentissi O (2019) Effects of seclusion and restraint in adult psychiatry: a systematic review. Front Psychiatry 10:491 31404294 10.3389/fpsyt.2019.00491 PMC6673758

[5] Gaskin C (2013) Reducing restrictive interventions: literature review and document analysis. Department of Health and Human Services, Melbourne, Vic

[6] Franks ZM, Alcock JA, Lam T, Haines KJ, Arora N, Ramanan M (2021) Physical restraints and post-traumatic stress disorder in survivors of critical illness. A systematic review and meta-analysis. Annals Am Thorac Soc 18(4):689–697 10.1513/AnnalsATS.202006-738OC 33075240

[7] Butterworth H, Wood L, Rowe S (2022) Patients’ and staff members’ experiences of restrictive practices in acute mental health in-patient settings: systematic review and thematic synthesis. BJPsych Open 8(6):e178 36200350 10.1192/bjo.2022.574 PMC9634587

[8] De Benedictis L, Dumais A, Sieu N, Mailhot M-P, Létourneau G, Tran M-AM, Stikarovska I, Bilodeau M, Brunelle S, Côté G (2011) Staff perceptions and organizational factors as predictors of seclusion and restraint on psychiatric wards. Psychiatric Serv 62(5):484–491 10.1176/ps.62.5.pss6205_0484 21532073

[9] World Health Organization (2019) Strategies to end seclusion and restraint: WHO quality rights specialized training: course guide

[10] Fletcher J, Hamilton B, Kinner S, Sutherland G, King K, Tellez JJ, Harvey C, Brophy L (2019) Working towards least restrictive environments in acute mental health wards in the context of locked door policy and practice. Int J Ment Health Nurs 28(2):538–550 30516024 10.1111/inm.12559

[11] Noorthoorn EO, Voskes Y, Janssen WA, Mulder CL, van de Sande R, Nijman HL, Smit A, Hoogendoorn AW, Bousardt A, Widdershoven GA (2016) Seclusion reduction in Dutch mental health care: did hospitals meet goals? Psychiatric Serv 67(12):1321–1327 10.1176/appi.ps.201500414 27364814

[12] Staggs VS (2015) Trends in use of seclusion and restraint in response to injurious assault in psychiatric units in US hospitals, 2007–2013. Psychiatric Serv 66(12):1369–1372 10.1176/appi.ps.201400490 26174946

[13] Hansen A, Hazelton M, Rosina R, Inder K (2020) Factors associated with seclusion use in forensic mental health settings: an integrative review. Int J Forensic Mental Health 19(2):198–213

[14] Roper C, McSherry B, Brophy L (2015) Defining seclusion and restraint: legal and policy definitions versus consumer and carer perspectives. J Law Med 23(2):297–302 26939495

[15] Belayneh Z, Chavulak J, Lee DCA, Petrakis M, Haines TP (2024) Prevalence and variability of restrictive care practice use (physical restraint, seclusion and chemical restraint) in adult mental health inpatient settings: a systematic review and meta-analysis. J Clin Nurs 10.1111/jocn.17041 38304928

[16] Adams HL, Matson JL Scope and prevalence of the problem. Comorbid Conditions Individuals Intellect Disabil 2015:3–24

[17] Danda MC (2022) Exploring the complexity of acute inpatient mental health nurses experience of chemical restraint interventions: implications on policy, practice and education. Arch Psychiatr Nurs 39:28–36 35688541 10.1016/j.apnu.2022.03.002

[18] Beck NC, Durrett C, Stinson J, Coleman J, Stuve P, Menditto A (2008) Trajectories of seclusion and restraint use at a state psychiatric hospital. Psychiatric Serv 59(9):1027–1032 10.1176/ps.2008.59.9.1027 18757596

[19] Ayuningtyas D, Rayhani M, Misnaniarti M, Maulidya AN (2018) Implementation of mental health policies toward Indonesia free restraint. Policy Gov Rev 2(2):161–173

[20] Vandamme A, Wullschleger A, Garbe A, Cole C, Heinz A, Bermpohl F, Mielau J, Mahler L, Montag C (2021) The role of implicit and explicit staff attitudes in the use of coercive measures in psychiatry. Front Psychiatry 12:699446 34220595 10.3389/fpsyt.2021.699446 PMC8249742

[21] Bowers L (2000) The expression and comparison of ward incident rates. Issues Ment Health Nurs 21(4):365–374 11249355 10.1080/016128400247988

[22] Knott J, Gerdtz M, Dobson S, Daniel C, Graudins A, Mitra B, Bartley B, Chapman P (2020) Restrictive interventions in victorian emergency departments: a study of current clinical practice. Emerg Med Australasia 32(3):393–400 10.1111/1742-6723.13412 31773838

[23] Fletcher J, Hamilton B, Kinner SA, Brophy L (2019) Safewards impact in inpatient mental health units in Victoria, Australia: staff perspectives. Front Psychiatry 462 10.3389/fpsyt.2019.00462 PMC6635577 31354541

[24] Staggs VS (2020) Variability in psychiatric facility seclusion and restraint rates as reported on hospital compare site. Psychiatr Serv 71(9):893–898 32487008 10.1176/appi.ps.202000011

[25] Janssen W, van de Sande R, Noorthoorn E, Nijman H, Bowers L, Mulder C, Smit A, Widdershoven GA, Steinert T (2011) Methodological issues in monitoring the use of coercive measures. Int J Law Psychiatry 34(6):429–438 22079087 10.1016/j.ijlp.2011.10.008

[26] Strack KM, Schulenberg SE (2009) Understanding empowerment, meaning, and perceived coercion in individuals with serious mental illness. J Clin Psychol 65(10):1137–1148 19670431 10.1002/jclp.20607

[27] Zhang Y, Akl EA, Schünemann HJ (2019) Using systematic reviews in guideline development: the GRADE approach. Res Synthesis Methods 10(3):312–329 10.1002/jrsm.1313 30006970

[28] Barr L, Wynaden D, Heslop K (2019) Promoting positive and safe care in forensic mental health inpatient settings: evaluating critical factors that assist nurses to reduce the use of restrictive practices. Int J Ment Health Nurs 28(4):888–898 30916443 10.1111/inm.12588

[29] Ruano J, Gómez-García F, Gay-Mimbrera J, Aguilar-Luque M, Fernández-Rueda JL, Fernández-Chaichio J, Alcalde-Mellado P, Carmona-Fernandez PJ, Sanz-Cabanillas JL, Viguera-Guerra I (2018) Evaluating characteristics of PROSPERO records as predictors of eventual publication of non-cochrane systematic reviews: a meta-epidemiological study protocol. Syst Reviews 7(1):1–6 10.1186/s13643-018-0709-6 PMC5845292 29523200

[30] Eldawlatly A, Alshehri H, Alqahtani A, Ahmad A, Al-Dammas F, Marzouk A (2018) Appearance of Population, intervention, comparison, and Outcome as research question in the title of articles of three different anesthesia journals: a pilot study. Saudi J Anaesth 12(2):283 29628841 10.4103/sja.SJA_767_17 PMC5875219

[31] Beckman M, Hensel N (2009) Making explicit the implicit: defining undergraduate research. CUR Q 29(4):40–44

[32] Hale B, Wright C Implicit definition and the a priori. New Essays priori 2000:286–319

[33] Piccinini G (2011) Two kinds of concept: implicit and explicit. Dialogue: Can Philosophical Review/Revue canadienne de philosophie 50(1):179–193

[34] Giovannini EN, Schiemer G (2021) What are implicit definitions? *Erkenntnis*. 86:1661–1691

[35] Hotzy F, Mötteli S, Theodoridou A, Schneeberger AR, Seifritz E, Hoff P, Jäger M (2018) Clinical course and prevalence of coercive measures: an observational study among involuntarily hospitalised psychiatric patients. Swiss Med Wkly 148:w14616 29698543 10.4414/smw.2018.14616

[36] Kyngäs H Inductive content analysis. Application Content Anal Nurs Sci Res 2020:13–21

[37] Vears DF, Gillam L (2022) Inductive content analysis: a guide for beginning qualitative researchers. Focus Health Prof Education: Multi-disciplinary J 23(1):111–127

[38] Di Lorenzo R, Baraldi S, Ferrara M, Mimmi S, Rigatelli M (2012) Physical restraints in an Italian psychiatric ward: clinical reasons and staff organization problems. Perspect Psychiatr Care 48(2):95–107 22458723 10.1111/j.1744-6163.2011.00308.x

[39] Flammer E, Eisele F, Hirsch S, Steinert T (2022) Increase in coercive measures in psychiatric hospitals in Germany during the COVID-19 pandemic. PLoS ONE 17(8):e0264046 36044407 10.1371/journal.pone.0264046 PMC9432719

[40] Lindgren BM, Ringnér A, Molin J, Graneheim UH (2019) Patients’ experiences of isolation in psychiatric inpatient care: insights from a meta-ethnographic study. Int J Ment Health Nurs 28(1):7–21 29975446 10.1111/inm.12519

[41] Askew L, Fisher P, Beazley P (2020) Being in a seclusion room: the forensic psychiatric inpatients’ perspective. J Psychiatr Ment Health Nurs 27(3):272–280 31755614 10.1111/jpm.12576

[42] Cole C, Vandamme A, Bermpohl F, Czernin K, Wullschleger A, Mahler L (2020) Correlates of seclusion and restraint of patients admitted to psychiatric inpatient treatment via a German emergency room. J Psychiatr Res 130:201–206 32829167 10.1016/j.jpsychires.2020.07.033

[43] Husum TL, Bjørngaard JH, Finset A, Ruud T (2010) A cross-sectional prospective study of seclusion, restraint and involuntary medication in acute psychiatric wards: patient, staff and ward characteristics. BMC Health Serv Res 10:1–9 20370928 10.1186/1472-6963-10-89 PMC2858144

[44] Jacob T, Sahu G, Frankel V, Homel P, Berman B, McAfee S (2016) Patterns of restraint utilization in a community hospital’s psychiatric inpatient units. Psychiatr Q 87:31–48 25899518 10.1007/s11126-015-9353-7

[45] Jegede OO, Ahmed SF, Olupona T, Akerele E (2017) Restraints utilization in a psychiatric emergency room. Int J Mental Health 46(2):125–132

[46] Mah TM, Hirdes JP, Heckman G, Stolee P (2015) Use of control interventions in adult in-patient mental health services. Healthcare Management Forum: 2015. SAGE Publications Sage CA, Los Angeles, CA, pp 139–145 10.1177/0840470415581230 26015489

[47] Amer AB (2013) Informed consent in adult psychiatry. Oman Med J 28(4):228 23904913 10.5001/omj.2013.67 PMC3725243

[48] Kokanović R, Brophy L, McSherry B, Flore J, Moeller-Saxone K, Herrman H (2018) Supported decision-making from the perspectives of mental health service users, family members supporting them and mental health practitioners. Australian New Z J Psychiatry 52(9):826–833 29952217 10.1177/0004867418784177

[49] Goulet M-H, Larue C, Dumais A (2017) Evaluation of seclusion and restraint reduction programs in mental health: a systematic review. Aggress Violent Beh 34:139–146

[50] Hem MH, Gjerberg E, Husum TL, Pedersen R (2018) Ethical challenges when using coercion in mental healthcare: a systematic literature review. Nurs Ethics 25(1):92–110 26931767 10.1177/0969733016629770

[51] Flammer E, Hirsch S, Steinert T (2021) Effect of the introduction of immediate judge’s decisions in 2018 on the use of coercive measures in psychiatric hospitals in Germany: a population-based study. Lancet Reg Health–Europe 11 10.1016/j.lanepe.2021.100233 PMC8577163 34778858

[52] Sampogna G, Luciano M, Del Vecchio V, Pocai B, Palummo C, Fico G, Giallonardo V, De Rosa C, Fiorillo A (2019) Perceived coercion among patients admitted in psychiatric wards: Italian results of the EUNOMIA study. Front Psychiatry 10:316 31164841 10.3389/fpsyt.2019.00316 PMC6536685

[53] Raboch J, Kališová L, Nawka A, Kitzlerová E, Onchev G, Karastergiou A, Magliano L, Dembinskas A, Kiejna A, Torres-Gonzales F (2010) Use of coercive measures during involuntary hospitalization: findings from ten European countries. Psychiatric Serv 61(10):1012–1017 10.1176/ps.2010.61.10.1012 20889640

[54] Asher L, Fekadu A, Teferra S, De Silva M, Pathare S, Hanlon C (2017) I cry every day and night, I have my son tied in chains: physical restraint of people with schizophrenia in community settings in Ethiopia. Globalization Health 13:1–14 28693614 10.1186/s12992-017-0273-1 PMC5504711

[55] Johnston MS, Kilty JM (2016) It’s for their own good: techniques of neutralization and security guard violence against psychiatric patients. Punishm Soc 18(2):177–197

[56] Hall JE, Karch DL, Crosby A (2016) Uniform definitions and recommended core data elements for use in elder abuse surveillance. Version 1.0

[57] Steinert T, Lepping P (2009) Legal provisions and practice in the management of violent patients. A case vignette study in 16 European countries. Eur Psychiatry 24(2):135–141 18455912 10.1016/j.eurpsy.2008.03.002

[58] Runciman W, Hibbert P, Thomson R, Van Der Schaaf T, Sherman H, Lewalle P (2009) Towards an international classification for Patient Safety: key concepts and terms. Int J Qual Health Care 21(1):18–26 19147597 10.1093/intqhc/mzn057 PMC2638755

[59] Belayneh Z, Abebaw D, Amare T, Haile K, Abebe Z (2019) Perception regarding the causes of schizophrenia and associated factors among Feresbet district residents: a community based study. BMC Public Health 19:1–7 30909977 10.1186/s12889-019-6678-4 PMC6434636

[60] De Cangas JP (1993) Nursing staff and unit characteristics: do they affect the use of seclusion? Perspect Psychiatr Care 29(3):15–22 8108229 10.1111/j.1744-6163.1993.tb00416.x

[61] Husum TL, Bjørngaard JH, Finset A, Ruud T (2010) A cross-sectional prospective study of seclusion, restraint and involuntary medication in acute psychiatric wards: patient, staff and ward characteristics. BMC Health Serv Res 10(1):1–9 20370928 10.1186/1472-6963-10-89 PMC2858144

[62] Riahi S, Thomson G, Duxbury J (2016) An integrative review exploring decision-making factors influencing mental health nurses in the use of restraint. J Psychiatr Ment Health Nurs 23(2):116–128 26809740 10.1111/jpm.12285

[63] Hempeler C, Braun E, Faissner M, Gather J, Scholten M (2024) Preferences of Individual Mental Health Service users are essential in determining the least restrictive type of Restraint. AJOB Neurosci 15(1):19–22 38207191 10.1080/21507740.2023.2292502

[64] Lawrence D, Bagshaw R, Stubbings D, Watt A (2022) Restrictive practices in adult secure mental health services: a scoping review. Int J Forensic Mental Health 21(1):68–88

[65] Robins LM, Lee D-CA, Bell JS, Srikanth V, Moehler R, Hill KD, Haines TP (2021) Definition and measurement of physical and chemical restraint in long-term care: a systematic review. Int J Environ Res Public Health 18(7):3639 33807413 10.3390/ijerph18073639 PMC8037562

[66] Wojt IR, Cairns R, Clough AJ, Tan EC (2021) The prevalence and characteristics of psychotropic-related hospitalizations in older people: a systematic review and meta-analysis. J Am Med Dir Assoc 22(6):1206–1214 e1205 33539820 10.1016/j.jamda.2020.12.035

[67] Semahegn A, Torpey K, Manu A, Assefa N, Tesfaye G, Ankomah A (2020) Psychotropic medication non-adherence and its associated factors among patients with major psychiatric disorders: a systematic review and meta-analysis. Syst Reviews 9:1–18 10.1186/s13643-020-1274-3 PMC6966860 31948489

[68] Hankin CS, Bronstone A, Koran LM (2011) Agitation in the inpatient psychiatric setting: a review of clinical presentation, burden, and treatment. J Psychiatric Practice^®^ 17(3):170–185 21586995 10.1097/01.pra.0000398410.21374.7d

[69] Muir-Cochrane E (2020) A wicked problem: Chemical restraint: towards a definition. Int J Ment Health Nurs 29(6):1272–1274 32888233 10.1111/inm.12780

[70] Mason T (1992) Seclusion: definitional interpretations. J Forensic Psychiatry 3(2):261–270

[71] O’Donovan D, Boland C, Carballedo A Current trends in restrictive interventions in psychiatry: a European perspective. BJPsych Adv 2022:1–9

[72] Huckshorn KA (2004) Reducing seclusion & restraint use in mental health settings: core strategies for prevention. J PsychoSoc Nurs Ment Health Serv 42(9):22–33 15493493 10.3928/02793695-20040901-05

[73] Haugom W, Ruud E, Hynnekleiv T (2019) Ethical challenges of seclusion in psychiatric inpatient wards: a qualitative study of the experiences of Norwegian mental health professionals. BMC Health Serv Res 19:1–12 31752958 10.1186/s12913-019-4727-4 PMC6873436

[74] Bendall C, Williams C, Huddy V (2022) Exploring experiences of restrictive practices within inpatient mental healthcare from the perspectives of patients and staff. J Psychiatric Intensive Care 18(1):17–29

[75] Hallett N, McLaughlin P (2022) Restrictive interventions: understanding and reducing their use in mental health settings. Mental Health Pract 25(6)

[76] Harris GT, Rice ME, Preston DL (1989) Staff and patient perceptions of the least restrictive alternatives for the short-term control of disturbed behavior. J Psychiatry Law 17(2):239–263

