# Supplementary material for: Variations in definitions used for describing restrictive care practices (seclusion and restraint) in adult mental health inpatient units: a systematic review and content analysis
Source: Soc Psychiatry Psychiatr Epidemiol. 2024 Jul 30;60(1):1–24. doi: 10.1007/s00127-024-02739-6 (PMC11790767; doi:10.1007/s00127-024-02739-6)
Supplement: Supplementary file 1 — Supplementary Material 1 [file 127_2024_2739_MOESM1_ESM.docx]

**Supplementary file 1: Concepts and key-terms used in the search strategies of Ovid databases, following the PIC approach**

| Inpatient/hospital and synonymous: **Population** | Restrictive care practices/ interventions and synonymous: **Intervention** | Mental health/Mental illness and synonymous: **Concept/Context** |
| --- | --- | --- |
| "In#patient*"  "Admitted patient*"  "Admission*"  "Hospital*"  "Ward*"  "Acute care*"  "Emergenc*"  "Intensive care"  "Clinic*" | "Restrictive practice*"  "Restrictive care"  "Restrictive intervention*"  "Restraint*"  "Seclusion*"  "Patient isolation"  "coercion" | "Mental health"  "Mental illness*"  "Mental disorder*"  "Mental disease"  "Psychiatr*"  "Psychotic"  "Psychosis"  "Schizophrenia"  "Bi#polar disorder*"  "Depression"  "Anxiety disorder*"  "Post#traumatic stress disorder"  "Mentally ill person*"  "People with mental illness" |

((("In?patient*" OR "Admitted patient*" OR "Admission*" OR "Hospital*" OR "Ward*" OR "Acute care*" OR "Emergenc*" OR "Intensive care" OR "Clinic*") AND ("Restrictive practic*" OR "Restrictive care" OR "Restrictive intervention*" OR "Restraint*" OR "Seclusion*" OR "Patient isolation" OR "Coercion")) AND ("Mental health" OR "Mental illness*" OR "Mental disorder*" OR "Mental disease" OR "Psychiatr*" OR "Psychotic" OR "Psychosis" OR "Schizophrenia" OR "Bi?polar disorder*" OR "Depression" OR "Anxiety disorder*" OR "Posttraumatic stress disorder" OR "Mentally ill person*" OR "People with mental illness")))

Limiters: -English language

-Publication years starting from 2010
