# Supplementary material for: Variations in definitions used for describing restrictive care practices (seclusion and restraint) in adult mental health inpatient units: a systematic review and content analysis
Source: Soc Psychiatry Psychiatr Epidemiol. 2024 Jul 30;60(1):1–24. doi: 10.1007/s00127-024-02739-6 (PMC11790767; doi:10.1007/s00127-024-02739-6)
Supplement: Supplementary file 2 — Supplementary Material 2 [file 127_2024_2739_MOESM2_ESM.docx]

**Supplementary file 2: Reference lists of all studies included in this systematic review**

1. Anderson E, Mohr DC, Regenbogen I, Swamy L, Smith EG, Mourra S, Rinne ST: **Influence of organizational climate and clinician morale on seclusion and physical restraint use in inpatient psychiatric units**. *Journal of Patient Safety* 2021, **17**(4):316-322.

2. An F-R, Sha S, Zhang Q-E, Ungvari GS, Ng CH, Chiu HF, Wu P-P, Jin X, Zhou J-S, Tang Y-L: **Physical restraint for psychiatric patients and its associations with clinical characteristics and the National Mental Health Law in China**. *Psychiatry research* 2016, **241**:154-158.

3. Barnett BS: **Factors associated with the use of seclusion in an inpatient psychiatric unit in Lilongwe, Malawi**. *Malawi medical journal* 2018, **30**(3):197-204.

4. Beaglehole B, Beveridge J, Campbell-Trotter W, Frampton C: **Unlocking an acute psychiatric ward: the impact on unauthorised absences, assaults and seclusions**. *BJPsych bulletin* 2017, **41**(2):92-96.

5. Bergk J, Flammer E, Steinert T: **" Coercion Experience Scale"(CES)-validation of a questionnaire on coercive measures**. *BMC psychiatry* 2010, **10**(1):1-10.

6. Bilanakis N, Kalampokis G, Christou K, Peritogiannis V: **Use of coercive physical measures in a psychiatric ward of a general hospital in Greece**. *Int J Soc Psychiatry* 2010, **56**(4):402-411.

7. Bilanakis N, Papamichael G, Peritogiannis V: **Chemical restraint in routine clinical practice: a report from a general hospital psychiatric ward in Greece**. *Annals of general psychiatry* 2011, **10**(1):1-3.

8. Bowers L, Ross J, Nijman H, Muir‐Cochrane E, Noorthoorn E, Stewart D: **The scope for replacing seclusion with time out in acute inpatient psychiatry in England**. *Journal of Advanced Nursing* 2012, **68**(4):826-835.

9. Brady NS, Spittal MJ, Brophy LM, Harvey CA: **Patients’ experiences of restrictive interventions in Australia: Findings from the 2010 Australian survey of psychosis**. *Psychiatric Services* 2017, **68**(9):966-969.

10. Bullock R, McKenna B, Kelly T, Furness T, Tacey M: **When reduction strategies are put in place and mental health consumers are still secluded: An analysis of clinical and sociodemographic characteristics**. *International Journal of Mental Health Nursing* 2014, **23**(6):506-512.

11. Chavulak J, Petrakis M: **Who experiences seclusion? An examination of demographics and duration in a public acute inpatient mental health service**. *Soc Work Health Care* 2017, **56**(6):524-540.

12. Chiba G, Subramaney U: **Is the seclusion policy of mental healthcare users a necessary evil?** *South African Journal of Bioethics and Law* 2015, **8**(1):30-34.

13. Chieze M, Courvoisier D, Kaiser S, Wullschleger A, Hurst S, Bardet-Blochet A, Ourahmoune A, Sentissi O: **Prevalence and risk factors for seclusion and restraint at Geneva’s adult psychiatric hospital in 2017**. *The European Journal of Psychiatry* 2021, **35**(1):24-32.

14. Cole C, Vandamme A, Bermpohl F, Czernin K, Wullschleger A, Mahler L: **Correlates of seclusion and restraint of patients admitted to psychiatric inpatient treatment via a German emergency room**. *Journal of Psychiatric Research* 2020, **130**:201-206.

15. Di Lorenzo R, Baraldi S, Ferrara M, Mimmi S, Rigatelli M: **Physical restraints in an Italian psychiatric ward: clinical reasons and staff organization problems**. *Perspectives in psychiatric care* 2012, **48**(2):95-107.

16. Danielsen A, Fenger M, Østergaard S, Nielbo K, Mors O: **Predicting mechanical restraint of psychiatric inpatients by applying machine learning on electronic health data**. *Acta Psychiatrica Scandinavica* 2019, **140**(2):147-157.

17. De Hert M, Einfinger G, Scherpenberg E, Wampers M, Peuskens J: **The prevention of deep venous thrombosis in physically restrained patients with schizophrenia**. *International journal of clinical practice* 2010, **64**(8):1109-1115.

18. Dumais A, Larue C, Drapeau A, Ménard G, Giguere Allard M: **Prevalence and correlates of seclusion with or without restraint in a Canadian psychiatric hospital: a 2‐year retrospective audit**. *Journal of psychiatric and mental health nursing* 2011, **18**(5):394-402.

19. Duxbury J, Baker J, Downe S, Jones F, Greenwood P, Thygesen H, McKeown M, Price O, Scholes A, Thomson G: **Minimising the use of physical restraint in acute mental health services: the outcome of a restraint reduction programme (‘REsTRAIN YOURSELF’)**. *International journal of nursing studies* 2019, **95**:40-48.

20. El-Abidi K, Moreno-Poyato AR, Privat AT, Martinez DC, Aceña-Domínguez R, Pérez-Solà V, Mané A: **Determinants of mechanical restraint in an acute psychiatric care unit**. *World Journal of Psychiatry* 2021, **11**(10):854.

21. Feeney L, Bonner N, McAndrew J: **Restrictive interventions on a psychiatric admission ward before and after COVID-19**. *Irish Journal of Psychological Medicine* 2022:1-7.

22. Flammer E, Eisele F, Hirsch S, Steinert T: **Increase in coercive measures in psychiatric hospitals in Germany during the COVID-19 pandemic**. *PLoS one* 2022, **17**(8):e0264046.

23. Flammer E, Steinert T: **Association between restriction of involuntary medication and frequency of coercive measures and violent incidents**. *Psychiatric services* 2016, **67**(12):1315-1320.

24. Flammer E, Hirsch S, Steinert T: **Effect of the introduction of immediate judge's decisions in 2018 on the use of coercive measures in psychiatric hospitals in Germany: a population-based study**. *The Lancet Regional Health-Europe* 2021, **11**:100233.

25. Flammer E, Steinert T: **Involuntary medication, seclusion, and restraint in German psychiatric hospitals after the adoption of legislation in 2013**. *Frontiers in psychiatry* 2015, **6**:153.

26. Hotzy F, Moetteli S, Theodoridou A, Schneeberger AR, Seifritz E, Hoff P, Jäger M: **Clinical course and prevalence of coercive measures: an observational study among involuntarily hospitalised psychiatric patients**. *Swiss Medical Weekly* 2018, **148**:w14616.

27. Fukasawa M, Miyake M, Suzuki Y, Fukuda Y, Yamanouchi Y: **Relationship between the use of seclusion and mechanical restraint and the nurse-bed ratio in psychiatric wards in Japan**. *International Journal of Law and Psychiatry* 2018, **60**:57-63.

28. Georgieva I, Mulder CL, Whittington R: **Evaluation of behavioral changes and subjective distress after exposure to coercive inpatient interventions**. *BMC psychiatry* 2012, **12**(1):1-11.

29. Gowda GS, Lepping P, Noorthoorn EO, Ali SF, Kumar CN, Raveesh BN, Math SB: **Restraint prevalence and perceived coercion among psychiatric inpatients from South India: A prospective study**. *Asian journal of psychiatry* 2018, **36**:10-16.

30. Griffiths C, Roychowdhury A, Girardi A: **Seclusion: the association with diagnosis, gender, length of stay and HoNOS-secure in low and medium secure inpatient mental health service**. *The Journal of Forensic Psychiatry & Psychology* 2018, **29**(4):656-673.

31. Guzman‐Parra J, Aguilera‐Serrano C, Huizing E, Bono del Trigo A, Villagrán JM, García‐Sánchez JA, Mayoral‐Cleries F: **A regional multicomponent intervention for mechanical restraint reduction in acute psychiatric wards**. *Journal of psychiatric and mental health nursing* 2021, **28**(2):197-207.

32. Guzmán‐Parra J, Aguilera‐Serrano C, Huizing E, Bono del Trigo A, Villagrán JM, Hurtado Melero V, García‐Sanchez JA, Mayoral‐Cleries F: **Factors associated with prolonged episodes of mechanical restraint in mental health hospitalization units in Andalusia**. *J Psychiatr Ment Health Nurs* 2022.

33. Guzman‐Parra J, Garcia‐Sanchez JA, Pino‐Benitez I, Alba‐Vallejo M, Mayoral‐Cleries F: **Effects of a Regulatory Protocol for Mechanical Restraint and Coercion in a S panish Psychiatric Ward**. *Perspectives in Psychiatric Care* 2015, **51**(4):260-267.

34. Guzman-Parra J, Guzik J, Garcia-Sanchez JA, Pino-Benitez I, Aguilera-Serrano C, Mayoral-Cleries F: **Characteristics of psychiatric hospitalizations with multiple mechanical restraint episodes versus hospitalization with a single mechanical restraint episode**. *Psychiatry Research* 2016, **244**:210-213.

35. Haefner J, Dunn I, McFarland M: **A quality improvement project using verbal de-escalation to reduce seclusion and patient aggression in an inpatient psychiatric unit**. *Issues in mental health nursing* 2021, **42**(2):138-144.

36. Hendryx M, Trusevich Y, Coyle F, Short R, Roll J: **The distribution and frequency of seclusion and/or restraint among psychiatric inpatients**. *The Journal of Behavioral Health Services & Research* 2010, **37**(2):272-281.

37. Hilger H, von Beckerath O, Kröger K: **Prophylaxis of venous thromboembolism in physically restrained psychiatric patients**. *International Journal of Psychiatry in Clinical Practice* 2016, **20**(3):187-190.

38. Hirose N, Morita K, Nakamura M, Fushimi K, Yasunaga H: **Association between the duration of physical restraint and pulmonary embolism in psychiatric patients: A nested case–control study using a Japanese nationwide database**. *Archives of Psychiatric Nursing* 2021, **35**(5):534-540.

39. Huf G, Coutinho E, Adams C: **Physical restraints versus seclusion room for management of people with acute aggression or agitation due to psychotic illness (TREC-SAVE): a randomized trial**. *Psychological medicine* 2012, **42**(11):2265-2273.

40. Husum TL, Bjørngaard JH, Finset A, Ruud T: **A cross-sectional prospective study of seclusion, restraint and involuntary medication in acute psychiatric wards: patient, staff and ward characteristics**. *BMC Health Services Research* 2010, **10**(1):1-9.

41. Hu F, Muir‐Cochrane E, Oster C, Gerace A: **An examination of the incidence and nature of chemical restraint on adult acute psychiatric inpatient units in Adelaide, South Australia**. *International journal of mental health nursing* 2019, **28**(4):909-921.

42. Jacob T, Sahu G, Frankel V, Homel P, Berman B, McAfee S: **Patterns of restraint utilization in a community hospital’s psychiatric inpatient units**. *Psychiatric Quarterly* 2016, **87**(1):31-48.

43. Janssen W, Noorthoorn E, Nijman H, Bowers L, Hoogendoorn A, Smit A, Widdershoven G: **Differences in seclusion rates between admission wards: does patient compilation explain?** *Psychiatric Quarterly* 2013, **84**(1):39-52.

44. Jegede OO, Ahmed SF, Olupona T, Akerele E: **Restraints utilization in a psychiatric emergency room**. *International Journal of Mental Health* 2017, **46**(2):125-132.

45. Jayaram G, Samuels J, Konrad SS: **Prediction and prevention of aggression and seclusion by early screening and comprehensive seclusion documentation**. *Innovations in clinical neuroscience* 2012, **9**(7-8):30.

46. Jury A, Lai J, Tuason C, Koning A, Smith M, Boyd L, Swanson C, Fergusson D, Gruar A: **People who experience seclusion in adult mental health inpatient services: An examination of health of the nation outcome scales scores**. *International Journal of Mental Health Nursing* 2019, **28**(1):199-208.

47. Knutzen M, Bjørkly S, Eidhammer G, Lorentzen S, Mjøsund NH, Opjordsmoen S, Sandvik L, Friis S: **Mechanical and pharmacological restraints in acute psychiatric wards—Why and how are they used?** *Psychiatry research* 2013, **209**(1):91-97.

48. Kuppili PP, Vengadavaradan A, Bharadwaj B: **A Cross-Sectional Study to Assess the Frequency of Restraint, and Knowledge and Attitudes of the Caregivers of Patients Toward Restraint in a General Hospital Psychiatry Setting from South India**. *Indian Journal of Psychological Medicine* 2022:02537176211061304.

49. Lai J, Jury A, Long J, Fergusson D, Smith M, Baxendine S, Gruar A: **Variation in seclusion rates across New Zealand's specialist mental health services: Are sociodemographic and clinical factors influencing this?** *International Journal of Mental Health Nursing* 2019, **28**(1):288-296.

50. Laila NH, Mahkota R, Shivalli S, Bantas K, Krianto T: **Factors associated with pasung (physical restraint and confinement) of schizophrenia patients in Bogor regency, West Java Province, Indonesia 2017**. *BMC psychiatry* 2019, **19**(1):1-8.

51. Larue C, Dumais A, Boyer R, Goulet M-H, Bonin J-P, Baba N: **The experience of seclusion and restraint in psychiatric settings: perspectives of patients**. *Issues in Mental Health Nursing* 2013, **34**(5):317-324.

52. Lee SJ, Cox A, Whitecross F, Williams P, Hollander Y: **Sensory assessment and therapy to help reduce seclusion use with service users needing psychiatric intensive care**. *Journal of Psychiatric Intensive Care* 2010, **6**(2):83-90.

53. Leerbeck SM, Mainz J, Bøggild H: **Use of coercion at Danish psychiatric wards by day of week and time of day**. *Danish Medical Journal* 2017, **64**(8):A5395.

54. Lepping P, Masood B, Flammer E, Noorthoorn EO: **Comparison of restraint data from four countries**. *Social psychiatry and psychiatric epidemiology* 2016, **51**(9):1301-1309.

55. Lickiewicz J, Adamczyk N, Hughes PP, Jagielski P, Stawarz B, Makara-Studzińska M: **Reducing aggression in psychiatric wards using Safewards-A Polish study**. *Perspectives in psychiatric care* 2020.

56. Lykke J, Hjorthøj C, Thomsen CT, Austin SF: **Prevalence, predictors, and patterns of mechanical restraint use for inpatients with dual diagnosis**. *Perspectives in Psychiatric Care* 2019, **56**(1):20-27.

57. Mah TM, Hirdes JP, Heckman G, Stolee P: **Use of control interventions in adult in-patient mental health services**. In: *Healthcare Management Forum: 2015*: SAGE Publications Sage CA: Los Angeles, CA; 2015: 139-145.

58. McLeod M, King P, Stanley J, Lacey C, Cunningham R: **Ethnic disparities in the use of seclusion for adult psychiatric inpatients in New Zealand**. *The New Zealand Medical Journal* 2017, **130**(1454):30.

59. Miodownik C, Friger MD, Orev E, Gansburg Y, Reis N, Lerner V: **Clinical and demographic characteristics of secluded and mechanically restrained mentally ill patients: a retrospective study**. *Isr J Health Policy Res* 2019, **8**(1):1-8.

60. Nawka A, Kalisova L, Raboch J, Giacco D, Cihal L, Onchev G, Karastergiou A, Solomon Z, Fiorillo A, Del Vecchio V: **Gender differences in coerced patients with schizophrenia**. *BMC psychiatry* 2013, **13**(1):1-10.

61. Nakamura M, Yasunaga H, Haraguchi T, Ando S, Sugihara T, Horiguchi H, Ohe K, Matsuda S, Fushimi K: **Length of mechanical restraint following haloperidol injections versus oral atypical antipsychotics for the initial treatment of acute schizophrenia: a propensity-matched analysis from the Japanese diagnosis procedure combination database**. *Psychiatry research* 2013, **209**(3):412-416.

62. Noda T, Sugiyama N, Sato M, Ito H, Sailas E, Putkonen H, Kontio R, Joffe G: **Influence of patient characteristics on duration of seclusion/restrain in acute psychiatric settings in J apan**. *Psychiatry and Clinical Neurosciences* 2013, **67**(6):405-411.

63. Noorthoorn EO, Voskes Y, Janssen WA, Mulder CL, van de Sande R, Nijman HL, Smit A, Hoogendoorn AW, Bousardt A, Widdershoven GA: **Seclusion reduction in Dutch mental health care: did hospitals meet goals?** *Psychiatric Services* 2016, **67**(12):1321-1327.

64. O'Callaghan AK, Plunkett R, Kelly BD: **The association between perceived coercion on admission and formal coercive practices in an inpatient psychiatric setting**. *International journal of law and psychiatry* 2021, **75**:101680.

65. Odgaard AS, Kragh M, Roj Larsen E: **The impact of modified mania assessment scale (MAS-M) implementation on the use of mechanical restraint in psychiatric units**. *Nordic Journal of Psychiatry* 2018, **72**(8):549-555.

66. Pérez‐Revuelta JI, Torrecilla‐Olavarrieta R, García‐Spínola E, López‐Martín Á, Guerrero‐Vida R, Mongil‐San Juan JM, Rodríguez‐Gómez C, Pascual‐Paño JM, González‐Sáiz F, Villagrán‐Moreno JM: **Factors associated with the use of mechanical restraint in a mental health hospitalization unit: 8‐year retrospective analysis**. *Journal of Psychiatric and Mental Health Nursing* 2021, **28**(6):1052-1064.

67. Poloni N, Ielmini M, Caselli I, Lucca G, Isella C, Buzzi AE, Rizzo LRM, Gasparini A, Introini G, Callegari C: **The use of mechanical restraint in a psychiatric setting: an observational study**. *Journal of Psychopathology* 2020, **26**:284-289.

68. Prinsloo B, Noonan A: **Use of seclusion in a general hospital acute psychiatric unit**. *Irish journal of psychological medicine* 2010, **27**(4):184-188.

69. Raboch J, Kališová L, Nawka A, Kitzlerová E, Onchev G, Karastergiou A, Magliano L, Dembinskas A, Kiejna A, Torres-Gonzales F: **Use of coercive measures during involuntary hospitalization: findings from ten European countries**. *Psychiatric services* 2010, **61**(10):1012-1017.

70. Reitan SK, Helvik A-S, Iversen V: **Use of mechanical and pharmacological restraint over an eight-year period and its relation to clinical factors**. *Nordic Journal of Psychiatry* 2018, **72**(1):24-30.

71. Saeed H, Khan MS, Batool SM, Ladak AA, Karim N, Aftab R: **Need of physical and chemical restraints: Experiences at inpatient psychiatric ward in a tertiary care hospital in Karachi, Pakistan**. *Journal of the College of Physicians and Surgeons--Pakistan: JCPSP* 2019, **29**(5):486.

72. Sampogna G, Luciano M, Del Vecchio V, Pocai B, Palummo C, Fico G, Giallonardo V, De Rosa C, Fiorillo A: **Perceived coercion among patients admitted in psychiatric wards: Italian results of the EUNOMIA study**. *Frontiers in Psychiatry* 2019, **10**:316.

73. Shahpesandy H, Tye N, Hegarty A, Czechovska J, Kwentoh ML, Wood A: **Rapid tranquillisation of acutely disturbed and violent patients: a retrospective cohort examination of 24 patients on a psychiatric intensive care unit**. *Journal of Psychiatric Intensive Care* 2015, **11**(S1).

74. Shepherd N, Parker C, Arif N: **Pattern of rapid tranquillisation and restraint use in a central London mental health service**. *Journal of Psychiatric Intensive Care* 2015, **11**(2):78-83.

75. Silić A, Savić A, Čulo I, Kos S, Vukojević J, Brumen D, Ostojić D: **Approach to emergencies in schizophrenia in University Hospital" Vrapče"**. *Psychiatria Danubina* 2018, **30**(suppl. 4):203-207.

76. Smith CM, Turner NA, Thielman NM, Tweedy DS, Egger J, Gagliardi JP: **Association of Black race with physical and chemical restraint use among patients undergoing emergency psychiatric evaluation**. *Psychiatric Services* 2022, **73**(7):730-736.

77. Tyrer S, Beckley J, Goel D, Dennis B, Martin B: **Factors affecting the practice of seclusion in an acute mental health service in Southland, New Zealand**. *The psychiatrist* 2012, **36**(6):214-218.

78. Taylor K, Mammen K, Barnett S, Hayat M, Dosreis S, Gross D: **Characteristics of patients with histories of multiple seclusion and restraint events during a single psychiatric hospitalization**. *Journal of the American Psychiatric Nurses Association* 2012, **18**(3):159-165.

79. Terrell C, Brar K, Nuss S, El-Mallakh RS: **Resource Utilization with the Use of Seclusion and Restraint in a Dedicated Emergency Psychiatric Service**. *Southern Medical Journal* 2018, **111**(11):703-705.

80. Staggs VS: **Variability in psychiatric facility seclusion and restraint rates as reported on hospital compare site**. *Psychiatric Services* 2020, **71**(9):893-898.

81. Pérez‐Toribio A, Moreno‐Poyato AR, Lluch‐Canut T, Molina‐Martínez L, Bastidas‐Salvadó A, Puig‐Llobet M, Roldán‐Merino JF: **Relationship between nurses' use of verbal de‐escalation and mechanical restraint in acute inpatient mental health care: a retrospective study**. *International journal of mental health nursing* 2022, **31**(2):339-347.

82. Välimäki M, Lam YTJ, Hipp K, Cheng PYI, Ng T, Ip G, Lee P, Cheung T, Bressington D, Lantta T: **Physical restraint events in psychiatric hospitals in Hong Kong: a cohort register study**. *International Journal of Environmental Research and Public Health* 2022, **19**(10):6032.

83. Verlinde A, Noorthoorn E, Snelleman W, Van den Berg H, Snelleman–van der Plas M, Lepping P: **Seclusion and enforced medication in dealing with aggression: A prospective dynamic cohort study**. *European psychiatry* 2017, **39**:86-92.

84. Vruwink FJ, Mulder CL, Noorthoorn EO, Uitenbroek D, Nijman HL: **The effects of a nationwide program to reduce seclusion in the Netherlands**. *BMC psychiatry* 2012, **12**:1-4.

85. Vruwink FJ, Noorthoorn EO, Nijman HL, VanDerNagel JE, Hox JJ, Mulder CL: **Determinants of seclusion after aggression in psychiatric inpatients**. *Archives of Psychiatric Nursing* 2012, **26**(4):307-315.

86. Vruwink FJ, VanDerNagel JE, Noorthoorn EO, Nijman HL, Mulder CL: **“Disruptive Behavior” or “Expected Benefit” Are Rationales of Seclusion Without Prior Aggression**. *Frontiers in psychiatry* 2022, **13**:555.

87. Wu WW-K: **Psychosocial correlates of patients being physically restrained within the first 7 days in an acute psychiatric admission ward: retrospective case record review**. *Eas As A Psyc* 2015, **25**(2):47-57.

88. Whitecross F, Lee S, Bushell H, Kang M, Berry C, Hollander Y, Sonmez G, Rauchberger I: **Implementing a psychiatric behaviours of concern team can reduce restrictive intervention use and improve safety in inpatient psychiatry**. *Australasian Psychiatry* 2020, **28**(4):401-406.

89. Zhu XM, Xiang YT, Zhou JS, Gou L, Himelhoch S, Ungvari GS, Chiu HF, Lai KY, Wang XP: **Frequency of physical restraint and its associations with demographic and clinical characteristics in a Chinese psychiatric institution**. *Perspectives in Psychiatric Care* 2014, **50**(4):251-256.

90. Mark I, Bell D, Lewis W, Gleeson S, O'Brien A: **A retrospective cohort study evaluating demographic and clinical characteristics associated with use of seclusion in a London psychiatric intensive care unit**. *Journal of Psychiatric Intensive Care* 2022, **18**(1):7-16.

91. Cole C, Klotz E, Junghanss J, Oster A, Vandamme A, Bermpohl F, Mahler L: **Coercive measures in psychiatry – When do they occur and who is at risk?** *Journal of Psychiatric Research* 2023, **164**:315-321.

92. Flemmerer M, Bühling-Schindowski F, Baumgardt J, Bechdolf A: **Predictors of the use of restraint in inpatient psychiatric care among patients admitted via the emergency department**. *Journal of Psychiatric Research* 2023, **162**:37-43.

93. Linkhorst T, Birkeland SF, Gildberg FA, Mainz J, Torp-Pedersen C, Bøggild H: **Use of the least intrusive coercion at Danish psychiatric wards: A register-based cohort study of 131,632 first and subsequent coercive episodes within 35,812 admissions**. *International journal of law and psychiatry* 2022, **85**:101838.

94. Guzmán‐Parra J, Aguilera‐Serrano C, Huizing E, Bono del Trigo A, Villagrán JM, Hurtado Melero V, García‐Sanchez JA, Mayoral‐Cleries F: **Factors associated with prolonged episodes of mechanical restraint in mental health hospitalization units in Andalusia**. *Journal of Psychiatric and Mental Health Nursing* 2022, **29**(6):873-882.

95. De Cuyper K, Vanlinthout E, Vanhoof J, van Achterberg T, Opgenhaffen T, Nijs S, Peeters T, Put J, Maes B, Van Audenhove C: **Best practice recommendations on the application of seclusion and restraint in mental health services: An evidence, human rights and consensus‐based approach**. *Journal of Psychiatric and Mental Health Nursing* 2023, **30**(3):580-593.
